# Aerobic Training and Mobilization Early Post-stroke: Cautions and Considerations

**DOI:** 10.3389/fneur.2019.01187

**Published:** 2019-11-15

**Authors:** Susan Marzolini, Andrew D. Robertson, Paul Oh, Jack M. Goodman, Dale Corbett, Xiaowei Du, Bradley J. MacIntosh

**Affiliations:** ^1^KITE, Toronto Rehab-University Health Network, Toronto, ON, Canada; ^2^Department of Exercise Sciences, Faculty of Kinesiology and Physical Education, University of Toronto, Toronto, ON, Canada; ^3^Canadian Partnership for Stroke Recovery, Toronto, ON, Canada; ^4^Schlegel-University of Waterloo Research Institute for Aging, University of Waterloo, Waterloo, ON, Canada; ^5^Department of Kinesiology, University of Waterloo, Waterloo, ON, Canada; ^6^Department of Cellular and Molecular Medicine, University of Ottawa, Ottawa, ON, Canada; ^7^School of Kinesiology and Health Studies, Queen's University, Kingston, ON, Canada; ^8^Sunnybrook Health Sciences Center, Toronto, ON, Canada

**Keywords:** exercise, rehabilitation, mobilization, stroke, recovery

## Abstract

Knowledge gaps exist in how we implement aerobic exercise programs during the early phases post-stroke. Therefore, the objective of this review was to provide evidence-based guidelines for pre-participation screening, mobilization, and aerobic exercise training in the hyper-acute and acute phases post-stroke. In reviewing the literature to determine safe timelines of when to initiate exercise and mobilization we considered the following factors: arterial blood pressure dysregulation, cardiac complications, blood-brain barrier disruption, hemorrhagic stroke transformation, and ischemic penumbra viability. These stroke-related impairments could intensify with inappropriate mobilization/aerobic exercise, hence we deemed the integrity of cerebral autoregulation to be an essential physiological consideration to protect the brain when progressing exercise intensity. Pre-participation screening criteria are proposed and countermeasures to protect the brain from potentially adverse circulatory effects before, during, and following mobilization/exercise sessions are introduced. For example, prolonged periods of standing and static postures before and after mobilization/aerobic exercise may elicit blood pooling and/or trigger coagulation cascades and/or cerebral hypoperfusion. Countermeasures such as avoiding prolonged standing or incorporating periodic lower limb movement to activate the venous muscle pump could counteract blood pooling after an exercise session, minimize activation of the coagulation cascade, and mitigate potential cerebral hypoperfusion. We discuss patient safety in light of the complex nature of stroke presentations (i.e., type, severity, and etiology), medical history, comorbidities such as diabetes, cardiac manifestations, medications, and complications such as anemia and dehydration. The guidelines are easily incorporated into the care model, are low-risk, and use minimal resources. These and other strategies represent opportunities for improving the safety of the activity regimen offered to those in the early phases post-stroke. The timeline for initiating and progressing exercise/mobilization parameters are contingent on recovery stages both from neurobiological and cardiovascular perspectives, which to this point have not been specifically considered in practice. This review includes tailored exercise and mobilization prescription strategies and precautions that are not resource intensive and prioritize safety in stroke recovery.

## Introduction

Approximately 13.7 million strokes occur worldwide every year–almost 38,000 per day (1). About one third of strokes are fatal, and another third leave survivors with permanent disability. Animal studies show favorable effects of early aerobic exercise interventions, which take advantage of the optimal time window for neural repair (2). However, little is known about the efficacy and safety of mobilization and aerobic exercise for augmenting or prolonging neural repair in the hyper-acute (0–24 h) and acute phases (1–7 days) post-stroke in humans [see Table 1 for definitions of phases post-stroke (3)]. While initiating exercise earlier in recovery may be beneficial, there is little evidence to justify the safety of early interventions with respect to neurobiological changes that could impact stroke volume, cell death, inflammation, or oxidative stress. Indeed, considerable preclinical evidence indicates it is not safe in the hyper-acute phase (4–7). Yet in clinical practice, patients are being mobilized within 12 h of admission, and aerobic training is being prescribed during in-patient rehabilitation (8–13) despite there being no guidelines for the safe prescription of intensity, duration, progression, and modality parameters during this time period (14).

**Table 1:** Timeframes for phase of stroke.

**Phase of stroke**	**Elapsed time from stroke onset**	
Hyper-acute	0–24 h	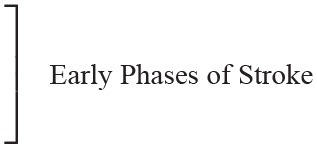
Acute	1–7 days	
Early subacute	7 days−3 months	
Late subacute	3–6 months	
Chronic	>6 months	

*Time frames have been adapted from Bernhardt et al. (3)*.

### Mobilization

Most contemporary stroke care guidelines and position papers advocate against “high-dose” or “intensive” out-of-bed activities within 24 h of stroke onset (15–19). The A Very Early Rehabilitation Trial (AVERT) played a key role as the results demonstrate a neutral or potentially negative effect of mobilization initiated within the first 24 h (20). Unfortunately, specific recommendations over and above the timing of the intervention are not provided in any set of guidelines. United Kingdom (UK) guidelines advise that mobilization within 24 h of onset should only be for patients who require little to no assistance (17). The guidelines further suggest that those with difficulty moving early after stroke, but who are medically stable, should be offered frequent, short daily mobilizations (sitting out of bed, standing, or walking), typically beginning between 24 and 48 h of stroke onset. Canadian guidelines advocate that frequent, out-of-bed activity within 24 h of stroke onset is not recommended, but that mobilization may be reasonable for some patients (18). Similarly, the National Institute for Health and Care Excellence guidelines recommend that, based in part on the committee's clinical experience, people who do not need help to sit out of bed, stand or walk, should be mobilized (sit, stand, or walk) in the first 24 h after symptom onset as the clinical condition permits, otherwise citing evidence suggesting that initiating high-intensity mobilization should not be offered in this time frame (21). Neither the UK nor Canadian guidelines defines what constitutes “high-dose,” “intensive,” or “frequent” mobilization. In addition, details on pre-participation health screening, contraindications to mobilization, and safe progression are minimal or absent. These recommendations have not considered the temporal biological changes occurring in the brain during recovery or how types of mobilization such as sitting, standing, and walking can affect these processes.

### Aerobic Exercise

Best practice guidelines are less clear in terms of aerobic exercise training. They indicate that given the potential benefits of aerobic exercise, little justification exists for not incorporating aerobic exercise into the care of the majority of cases once the individual is medically stable (22). They do acknowledge, however, a dearth of evidence regarding safety and effects of aerobic exercise prescribed in the acute phase post-stroke. As with mobilization, pre-participation screening criteria, cautions, considerations, and recommendations for intensity or other parameters of the exercise program in the hyper-acute and acute phases post-stroke represent gaps in knowledge.

Herein, we review the literature to advance consideration on the appropriate timing for the initiation of mobilization and aerobic exercise. We conduct a focused examination of the literature to determine the rate of recovery of arterial blood pressure (BP) dysregulation, cardiac complications, blood-brain barrier disruption, hemorrhagic stroke transformation, and ischemic penumbra viability. We contrast this to an estimate of when cerebral autoregulation (CA) is sufficiently restored so as to protect the brain from these possible disruptions that could be intensified with mobilization and aerobic exercise. We review the outcomes and methodology of studies that address the effects of mobilization and aerobic exercise in the hyper-acute and acute stages of stroke that help to inform a safety and efficacy framework. We also discuss countermeasures to protect the brain from exposure to potentially adverse circulatory effects before, during, and following exercise/mobilization sessions. Because neurobiological and cardiovascular recovery continues beyond the acute phase in some cases, our safety related recommendations may extend to the early subacute phase of recovery (7 days to 3 months).

## Peripheral and cerebral circulatory considerations for exercise and mobilization

Peripheral and cerebral circulatory changes that occur from the hyper-acute to early subacute phase post-stroke can leave the brain vulnerable to possible adverse effects of mobilization and physical activity-induced perturbations. Mobilization and aerobic exercise, depending on the intensity and type, result in rising noradrenaline and adrenaline plasma concentrations that increase systemic BP (23–25) that can be passed onto the vulnerable cerebral circulation. Within this context, awareness of the post-stroke status of CA, the blood brain barrier (BBB), and resting systemic BP regulation is critical. Hemorrhagic stroke warrants additional considerations, such as stroke progression and hematoma expansion following an intracerebral hemorrhage (ICH) and delayed ischemia and vasospasm following a subarachnoid hemorrhage (SAH). Insight into the progression and severity of these impairments can guide the timing of initiation, the intensity of activity, the level of implementation of the suggested strategies, and when the strategies can be gradually reduced or phased out.

### Cerebral Autoregulation Impairment Following Stroke

The importance of CA in the early phases post-stroke is evident from studies on final infarct size and neurological outcome (26–28). The classic view of CA is a static paradigm which describes the regulation of stable cerebral blood flow (CBF) over a wide range of perfusion pressures (~50–150 mmHg), although the nature of the CBF “plateau” and limits of BP within which CBF is regulated has recently come under scrutiny (29). In contrast, dynamic CA characterizes the cerebrovascular response to dynamic changes in blood pressure (30). Compelling evidence shows that CA can be impaired in the early phases following ischemic, intracerebral, and subarachnoid hemorrhagic strokes, and that restoration of normal CA function take up to 3 months post-stroke (26, 31–40). This implies that in the early phases post-stroke the brain may not be fully protected from fluctuations in BP that occur with mobilization or aerobic exercise. Poor CA appears to be associated with damage to the neurovascular unit and consequently threatens survival of neurons and glial cells (41, 42). While this sequelae is largely untested in humans, it is prudent to consider the clinical implications.

#### Ischemic Stroke and Cerebral Autoregulation

**Figure 1:**
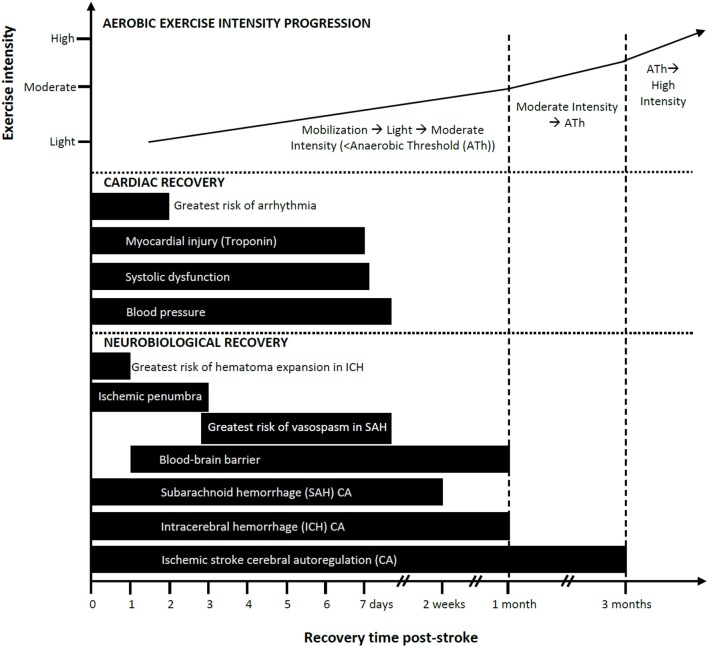
Progression of mobilization and aerobic exercise intensity in relation to estimated neurobiological and cardiac recovery post-stroke: a conceptual model. Aerobic exercise can ideally increase in intensity as a function of elapsed time post-stroke and should be guided based on cardiopulmonary fitness measures such as the anaerobic threshold (Ath). Safe and recommended periods to introduce exercise/mobilization post-stroke are shown here as varying by cardiac and neurobiological recoveries. Impaired cerebral autoregulation after ischemic stroke is listed here as the longest time to recovery. Recovery is based on available evidence.

Knowledge of the temporal profile of CA recovery would help in estimating when the brain is adequately protected from BP fluctuations associated with mobilization or exercise. Collectively, studies (described in detail in the Supplementary Materials) suggest impaired CA at baseline with worsening in the first 1–2 weeks and recovery by ~3 months post-ischemic stroke (see Figure 1). However, a limitation of the reviewed studies is the lack of measurements conducted between 1 and 3 months and thus recovery may occur earlier.

##### Association between cerebral autoregulation impairment and clinical outcomes

CA impairment contributes to poorer outcomes following ischemic stroke, including higher all-cause mortality and larger infarct size (26, 43). In a pooled analysis of two data sets (*n* = 45 ischemic stroke patients), impairment in CA around day 6 post-stroke was associated with poorer 4-month clinical outcome measured by the modified Rankin Scale (mRS) (27). In a separate study (*n* = 46), impaired CA very early (<6 h) post-stroke was associated with hemorrhagic transformation and cerebral edema at 24 h (28). Moreover, Castro et al. demonstrated that poorer efficacy of dynamic CA within 6 h of ischemic stroke resulted in larger infarct volumes at 24 h and poorer neurologic outcomes at 3 months measured by mRS (*n* = 30) (26). In fact, the odds of living independently (mRS 0–2) at 3 months were 14-fold higher when CA had recovered at 6 h post-stroke. This study was especially important as it suggests that impaired CA is not just a reflection of the severity of the stroke at baseline, but predicts adverse outcome independent of baseline National Institutes of Health Stroke Scale (NIHSS) score and age (26, 44).

##### Impaired cerebral autoregulation, hypotensive episodes, and the ischemic penumbra

The brain has less protection against acute episodes of hypotension than hypertension following stroke (type not specified) and brain injury (45–48). These findings are provocative and intriguing; they challenge a conventional safety concern of the hypertensive response when it comes to monitoring BP as a clinical indicator for safe exercise and mobilization. Preventing hypotensive episodes may be a greater safety concern than previously thought. Hypotension can occur after prolonged inactive standing and upon cessation of an exercise session (i.e., post-exercise hypotension). It is sensible to assume that the combination of reduced BP and poor CA can potentially foster hypoperfusion. The fate of the ischemic penumbral tissue surrounding the stroke core is one aspect of acute stroke management where low BP is a well-established hazard and for which transient hypotensive episodes could play a role. Whether the penumbral tissue succumbs to the necrotic core will depend on cerebral perfusion pressure and collateral supply (49). This potentially salvageable ischemic penumbra exists for at least 24 h post-stroke and can persist for days [for review (50–53)]. Compromised cells may recover if conditions are ideal, however hypotension, stress, and other challenges could cause their demise. While the effects of repeated hypotensive episodes related to activity (e.g., posture change, prolonged standing, post-dynamic exercise) have not been examined post-stroke, strategies to mitigate their occurrence should be considered. Precautionary guidelines are provided in **Tables 4–6**.

#### Intracerebral Hemorrhage and Cerebral Autoregulation

While fewer studies have assessed CA following ICH compared to ischemic stroke, the evidence suggests there is little to no CA impairment at baseline, worsening from days 3–12, and recovery by ~1 month post-ICH (see Figure 1; described in detail in the Supplementary Materials). A limitation is that there is a dearth of measurements conducted between days 12 and 30 thus recovery could occur earlier than 1 month.

##### Impaired cerebral autoregulation, association with clinical outcomes, and recovery time

While ICH is not associated with a penumbra at risk for further infarction (as in ischemic stroke) (69), hematoma expansion can occur in the first 24 h (as observed in 39 of 103 patients) (70) and may be exacerbated by less intense BP management (i.e., allowing higher BP) (71). This is likely mediated by lack of cerebral protection that under normal circumstances is offered by CA. Indeed, poorer CA is documented at 3–5 days post-ICH, and is associated with poor clinical status, ventricular hemorrhage, lower cerebral perfusion pressure, and worse functional recovery at 90 days (*n* = 26) (72). In a larger study (*n* = 43), impaired CA at days 4–6 was a predictor of poorer mRS at 90 days. This association was independent of the hematoma location, ICH volume, BP, neurological status (NIHSS), age, and sex (31). Given the adverse effects of higher systemic BP on hemorrhage expansion early post-ICH when CA is impaired, elevations in BP during mobilization and/or exercise could further exacerbate hematoma expansion.

#### Subarachnoid Hemorrhagic Stroke and Cerebral Autoregulation

The exact time course of CA recovery following SAH is not known. The available evidence (described in detail in the Supplementary Materials) suggests a recovery profile that features impairment through days 1–4 that gradually deteriorates, in some cases, before recovering by days 10–14 post-stroke (see Figure 1).

##### Association between impaired cerebral autoregulation and delayed ischemia and vasospasm

Otite et al. reported that of 68 patients with SAH, 62% developed angiographic vasospasm, and 19% had delayed cerebral ischemia on days 2–4 post-hospital admission (33). CA was impaired in those who developed vasospasm and delayed ischemia compared to those who did not, and was highly predictive of these adverse conditions. Indeed, consistent evidence indicates that dynamic CA is impaired post-SAH (38–40, 73–75), which is thought to play a role in delayed cerebral ischemia (76) and infarction after SAH (77–79). Loss of cerebral protection is clinically significant as vasospasm is a leading cause of morbidity and mortality after SAH and ischemia may occur when autoregulation does not compensate. Therefore, mobilization or exercise prescriptions that result in BP fluctuations should be considered carefully during days ~3–7 after aneurysmal SAH when there is elevated risk for delayed ischemia and vasospasm (33, 80, 81) and BP countermeasure strategies should be employed.

### Blood-Brain Barrier Disruption

The BBB protects neural tissue and the microenvironment by regulating the movement of molecules between blood and brain (82). BBB disruption allows proteins, cells, and large molecules to move from the lumen space into the brain parenchyma. The infiltration and accumulation of peripheral immune cells, pro-inflammatory cytokines, and an excess of water and other potentially toxic elements into the brain leads to progression of injury, cerebral edema, and increases the risk of hemorrhage following stroke (especially following tissue plasminogen activator (tPA) or delayed tPA treatment) (83, 84).

There appears to be two phases of BBB disruption after ischemic stroke (85, 86). As early as 2 h after ischemia in primates (87), and as early as 6 h in humans (88), the BBB has increased permeability. Early reperfusion can reverse BBB changes, but if reperfusion occurs later it may exacerbate endothelial injury. The second phase of BBB injury occurs within 24–72 h post-stroke and can result in greater tissue damage in humans (86). Low level BBB dysfunction has been detected up to 1 month following ischemic stroke (spontaneous reperfusion) in humans (89). BBB dysfunction is more likely to remain elevated in people with larger infarcts in the subacute phase (86). Animal studies also indicate that BBB function can take up to 3–4 weeks to recover post-ischemia with peak dysfunction at around 7 days (90–92) (see Figure 1).

Intensive exercise is documented to transiently induce hyperperfusion and cerebral edema, subsequent to mechanical disruption of the BBB in healthy and obese individuals (93–95). Although these physiological effects are temporary and not known to induce structural brain damage, possible adverse effects of higher intensity exercise may be of concern for up to ~1 month post-stroke in some patients. This is in part owing to BBB dysfunction and loss of its structural integrity occurring in people following ischemic and hemorrhagic stroke leaving the brain more vulnerable to damage (83, 86, 96).

While the effects of exercise on BBB function have not been measured in people following stroke, it is likely that CA dysfunction does not adequately counter the elevation in systemic BP during higher intensity exercise (95), thereby increasing the risk of cerebral hyperperfusion injury and BBB disruption (93). Consider also that superimposed on impaired CA and BBB dysfunction is elevated resting BP that occurs in up to 84% of stroke patients mostly in the acute phase post-stroke (97–100). This further challenges CA and BBB function to maintain homeostasis. Although there is a decrease in BP during the first 10 days following stroke, it remains elevated in about a third of cases (97–101). Thus, mobilization and aerobic exercise in the presence of elevated resting BP, impaired CA, and BBB disruption may theoretically interfere with the supply-demand relationship of cerebral oxygen delivery and ultimately contribute to deleterious hemodynamic effects.

Hydration status and environmental temperature are other factors that may exacerbate BBB dysfunction during exercise. Although there is some conflicting evidence (102), endurance exercise in a warm environment may lead to increased BBB permeability in healthy individuals (103, 104) and is likely related to dehydration and/or brain temperature. Heightened temperature in the first few days of stroke, due to mild fever or exercise, has the potential to exacerbate cell death which is still evolving at this time, contributing to poorer functional outcomes (105, 106). Thus, appropriate precautions should be practiced by ensuring the patient is hydrated before initiating activity and avoiding activity in a warm environment or during fever.

BBB disruption is generally considered detrimental post-stroke; however, in some cases increased permeability may be beneficial. For example, infiltrating macrophages post-ICH stroke may be involved in hematoma resolution (96) and certain types of leukocytes could be protective in ischemic stroke (107, 108). In addition, indirect evidence in obese and healthy populations suggests that exercise-induced BBB leakage detected may lead to acutely elevated levels of neurotrophic factors in the blood such as brain derived neurotrophic factor (BDNF) (94, 109) that would support neuronal survival and growth. However, the evidence of increased peripheral BDNF levels concomitant with evidence of BBB leakage in these exercise studies may be largely related to increased production through muscle action and restricted cerebral uptake, suggesting little to no benefit. Further studies are required to disentangle these effects.

### Effect of Age, Diabetes, and Hypertension on Blood-Brain Barrier Recovery and Cerebral Autoregulation

There is a rationale for delaying moderate to higher intensity exercise in the elderly, as well as those with persistent hypertension and/or diabetes/hyperglycemia (See Table 2 Guideline 1, Figure 1, and Supplementary Materials). CA impairment may be more problematic among stroke patients with comorbid diabetes, as suggested from observations of impaired CA in type II diabetes studies (116, 117) and higher mortality rates in those with hyperglycemia at the time of stroke (118). In addition, the time course of recovery of BBB function can be affected by age, hypertension and/or diabetes/hyperglycemia and should be considered when screening patients for initiating mobilization and aerobic exercise (119).

**Table 2:** Guideline 1.0: Pre-participation screening criteria based on peripheral and cerebral circulatory considerations.

This section provides blood pressure guidelines prior to early aerobic exercise (specifically hyper-acute and acute). A list of safe indications to consider are provided here: Consider either very light activity (following precautions in guidelines 3.0–6.0) or delaying aerobic exercise if resting systolic blood pressure is <120 mmHg, or higher than 170 mmHg. Consider either very light activity (following precautions in guidelines 3.0–6.0) or delaying aerobic exercise if resting diastolic blood pressure is <80 mmHg, or higher than 105 mmHg. A series of 4–10 resting blood pressures performed over the course of 1–3 days should be stable. The day to day variation in SBP should be <30%. Patients that are elderly, have diabetes/hyperglycemia, and/or persistent hypertension should be considered higher risk stroke subgroups thus it is advisable to delay moderate to higher intensity exercise post-stroke (see Figure 1). Consider delaying higher intensity exercise for people with blood glucose level of ≥160 mg/dL (≥9 mmol/L) measured within the first 48 h of stroke. There should be no evidence of dehydration prior to initiating activity. Warm environmental temperatures should be avoided and replacement of fluids recommended. Caution is warranted for those patients with the following conditions: anemia, early neurological deterioration, chest infection, and pulmonary emboli (110–115).	

*Patients should be screened on a case-by-case basis*.

### Blood Pressure

Elevated resting BP is common during acute stroke, thus should be a central consideration of exercise prescription. Current guidelines state aerobic exercise is not recommended post-stroke if the person has resting systolic (SBP) and diastolic BP (DBP) > 200 mmHg and >110 mmHg, respectively (22). These upper limits may be appropriate for people in the late subacute phase of stroke, but are less established in the early phases post-stroke given impaired CA and the effect on BBB integrity. A further activity-related elevation in BP from a resting SBP of 200 mmHg, is indeed a challenge to CA. Moreover, it may be prudent to establish a lower BP threshold, below which is a risk of hypoperfusion episodes. Until such data are available, we suggest a more conservative approach than is currently practiced. Even with more contemporary guidelines advocating for more tightly managed BP early in ischemic and ICH stroke (15, 120), it continues to be important to determine these thresholds for safe exercise. The Scandinavian Candesartan Acute Stroke Trial highlights the challenge of controlling BP. Ischemic or hemorrhagic stroke patients (n = 293) with a resting SBP of at least 140 mmHg were randomized to receive either candesartan or a placebo. BP at 7 days post-stroke was similar between the treatment and placebo group, with pressure in both groups remaining elevated (147/82 mmHg (SD 23/14) in the candesartan group and 152/84 mmHg (SD 22/14) in the placebo group) (101). Other studies report a mean decrease in BP during the first 10 days following stroke, but BP tends to remain elevated in one third of cases (97–100).

Hyper-acute and acute phases post-stroke are often characterized by BP instability. Within 24 h after stroke, SBP can decrease by 28 ± 11% either spontaneously or with medication (121). Increased systolic and/or diastolic ambulatory BP variability within 7 days of ischemic stroke has been associated with increased risk of recurrent stroke and composite cardiovascular endpoints, and poorer functional outcomes within 12 months of the stroke (122–124). Therefore, along with upper and lower BP boundaries, a maximal rate of change over 24 h, or a limit of BP variability (systolic and diastolic) that indicates stability, should be an additional screening criteria to ensure safe early exercise post-stroke (see Table 2 Guideline 1).

#### Monitoring BP in Advance of Aerobic Exercise and Mobilization: Screening Recommendations

While there is little evidence to support a specific BP threshold, there is a precedent for pre-activity BP screening criteria. A recent study in 708 post-ischemic stroke patients demonstrated increased odds of cognitive impairment at 3 months for patients in the lowest and highest systolic BP (SBP) quintiles within 7 days of stroke (Q1, 102–127 mmHg and Q5, 171–215 mmHg), relative to the middle quintile (Q3, 143–158 mmHg) after adjustment (55). Similarly, better outcomes were observed for patients in the middle quintile of diastolic BP (DBP) (Q3, 93–102 mmHg). This association continued for up to 6 months post-stroke. From a cardiac risk standpoint, baseline SBP < 110 mmHg predisposes people post-stroke to sudden cardiac adverse events (110). Further, lower early BP (DBP < 70, SBP < 155 mmHg) is a predictor of death within 90 days of acute ischemic stroke compared to those in the ranges of DBP 70–105 mmHg and SBP 155–220 mmHg (43) (see Table 2 Guideline 1).

## Cardiac Considerations for Exercise and Mobilization (see Table 3 Guideline 2)

Cardiovascular complications are a major cause of morbidity and mortality following stroke, and thus can affect the timing and intensity of the exercise prescription, as well as provide screening criteria particularly in the hyper-acute and acute phases post-stroke. Knowledge of the prevalence, time since first presentation, and other cardiac medical history information can guide the practitioner.

**Table 3:** Guideline 2.0: Cardiac screening criteria.

The goal following stroke is to initiate an exercise program as soon as the patient is clinically stable. Exercise should be prescribed with caution when initiated within 2 weeks post-stroke given that almost 2 out of every 10 patients experience an early serious cardiac adverse event. Adverse event occurrence peaks between day 2 and 3 post-stroke, with deaths from neurological and cardiac issues peaking during the second week.	
A patient is considered safe to initiate exercise if they satisfy the following criteria: No symptoms of coronary artery disease such as chest pain or shortness of breath in the past 24 h. ∙ No changes or normalization of the ECG in the past 12 h. No current significant ECG abnormalities such as frequent ventricular premature beats (≥3 in 10), or QT prolongation. No new signs of uncompensated heart failure in the previous 7 days. Troponin levels are normal within 3 days of stroke, or are normal 3–7 days following detection of elevated troponin levels. In patients with atrial fibrillation, systolic dysfunction, or other issues that reduce cardiac output, light intensity exercise should be maintained until these issues have resolved or until expected recovery of CA. Precautions for avoiding hypotensive episodes (orthostatic hypotension, prolonged standing, and post-exercise hypotension), should be followed during very early and early mobilization (see guidelines 3–5).	

*Cardiac-specific troponin measures and ECG monitoring are standard of care post-stroke at most institutions. Thus, the results should be reviewed prior to initiating exercise/mobilization following SAH, ischemic, and ICH stroke types especially in the hyper-acute and acute phases. ECG monitoring of people with insular strokes may be prudent. Delaying exercise in patients with elevated troponin with no evidence of CAD is recommended given the micro damage and associated ECG abnormalities and wall motion abnormalities*.

### Cardiac Complications and Morbidity and Mortality

Cardiac-related complications are the second leading cause of mortality within the first month after the stroke event (125, 126). Among 444 patients with first cerebral infarct, 17% died within the first month of the stroke (50% of deaths were due to the cerebral infarct, 12% due to cardiovascular events, and 38% for other reasons) (125). Of 980 patients with first ischemic stroke enrolled in the Northern Manhattan Study, 5% died in the first month post-stroke; the major cause of death at 55% were neurological causes, while 19% were cardiac (126). Prosser et al. revealed a more specific temporal profile of early cardiac morbidity and mortality (110). Of 846 patients followed during the first 3 months after acute ischemic stroke, the proportion of deaths due to neurological and cardiac causes were 43.9% (*n* = 79) and 19.4% (*n* = 35), respectively. Most of the neurologic deaths occurred in the first 2 weeks post-stroke, while cardiac deaths were highest in the second week. Furthermore, 19% (*n* = 161) of all patients experienced at least one serious cardiac adverse event within 3 months of the stroke that peaked in frequency between day 2 and 3 post-stroke. Cardiac complications included non-fatal arrhythmias, acute myocardial infarction, pulmonary edema/moderate-severe cardiac failure, and cardiac death.

### The Brain-Heart Connection

The high rate of cardiac manifestations following acute stroke highlights the brain-heart connection. Cardiac conditions that occur following stroke may be unrelated complications of stroke, or directly related to the underlying cause of the stroke such as atrial fibrillation in the case of cardioembolic stroke. Compelling evidence shows that brain damage is a causative factor in some cardiac conditions. The mechanistic basis underlying stroke-induced myocardial damage is complex and multi-factorial, potentially involving activation of the hypothalamic-pituitary-adrenal axis, dysregulation of the autonomic system, inflammation, gut microbiome dysbiosis, immune activation, and dysregulation of the autonomic nervous system and catecholamine “surge” (127). Catecholamine surge is associated with cardiac damage, myocardial stunning, an influx of inflammatory cells in the heart, and increased release of intracellular calcium ions and myocyte dysfunction (128–131). This is hypothesized to lead to ECG and structural cardiac changes even when there is no underlying heart disease. While, some of these stroke-induced changes can be mild or transient, some can be severe or potentially fatal.

### Effects of Stroke on the Heart

Effects of a stroke on the heart can include reduced ejection fraction, regional wall motion abnormalities, ECG changes, and arrhythmias (e.g., ventricular and supraventricular tachyarrhythmias, ST segment change, QT prolongation, tall and inverted *T* waves, and prominent *U* waves) and cardiac damage which can lead to chronic heart failure, as well as neurogenic stress-induced cardiomyopathy (most commonly transient left ventricular (LV) apical ballooning). Exercise therapists should have an understanding of the brain-heart interaction as there is a potential for exercise to interfere with recovery of cardiac function when introduced in the hyper-acute to acute phases post-stroke. Conversely, when cardiac function is compromised, early exercise may interfere with recovery of the brain. Specifically, the cardiac manifestations of stroke that reduce cardiac output that occur mostly in the hyper-acute and acute post-stroke phases can affect CBF when CA may be impaired. As demonstrated in a pre-clinical study of induced unilateral stroke, CBF becomes dependent on cardiac output in the absence of intact CA (132). Therefore, when autoregulatory control in the ischemic brain region is impaired early post-stroke, CBF is in part dependent on cardiac output in both positive and negative directions. This reliance on cardiac output has also been demonstrated in people with valvular disease where cardiac output is attenuated during exercise (133).

The clinical impact of cardiac output on the stroke brain is not well-established; compensatory responses to maintain cerebral oxygen metabolism, and the perfusion thresholds may be variable between human and animals (134). However, several studies have demonstrated that CBF is reduced in people with lower cardiac ejection fraction after stroke and in those with heart failure (135–137). One study showed that a change in posture from supine to upright resulted in a greater reduction in CBF-velocity in people with heart failure compared to an age- and sex-matched control group (138). Therefore, some of the cardiac manifestations following stroke can affect cardiac output and threaten perfusion to ischemic brain tissue (139). Initiating exercise in the presence of impaired CA superimposed on these cardiac abnormalities might compromise brain health. We suggest that people with systolic cardiac dysfunction (ventricular wall motion abnormalities and reduced ejection fraction), arrhythmias that compromise cardiac output, and elevated cardiac enzymes indicating cardiac damage maintain light activity/aerobic exercise until CA recovery and the cardiac complication is resolved and stable (see Table 3 Guideline 2.0 and Figure 1) (the rate of cardiac recovery is described in detail in the Supplementary Materials). While most studies report declines in cardiac contractile (systolic) performance, impaired diastolic dysfunction may also accompany declines in systolic function, particularly in patients diagnosed with neurogenic stress cardiomyopathy, which includes clinical symptoms of reduced LV ejection fraction, ventricular wall motion abnormalities, and elevated cardiac-specific serum enzymes (127).

#### Systolic Dysfunction and Poor Outcomes

Studies in consecutive hospital admissions for ischemic stroke demonstrated that between 13 and 29% of people had reduced LV systolic dysfunction (i.e., ejection fraction of <50%) (140–142). In SAH, depressed LV function and cardiac regional wall motion abnormalities are reported in 13–25% of cases. Although these complications are usually reversible, they are associated with high mortality, delayed cerebral ischemia, and poor functional outcomes (139, 143–148). A recent study examined SAH patients within 24 h of admission and found focal and global cerebral perfusion were significantly lower in 35 people with cardiac dysfunction (myocardial wall motion abnormality and/or positive cardiac troponin level) compared to 37 people without cardiac dysfunction (139). The authors point out that it is unknown if the link between cardiac dysfunction and cerebral perfusion is causal or if it is due to external causes that influence both cardiac function and cerebral perfusion such as a catecholamine surge. However, a recent preclinical study in focal cerebral ischemia demonstrated that increased sympathetic activity is a driver of the development of chronic systolic dysfunction (149).

Another link between systolic dysfunction and poor outcomes is the presence of low SBP at baseline in the acute phase. Prosser et al. have demonstrated that a baseline SBP of <110 mmHg predisposes people post-stroke to sudden cardiac adverse events (110). Stead et al. have reported that lower early BP (DBP < 70 or SBP < 155 mmHg) is a predictor of death within 90 days of acute ischemic stroke compared to those considered normotensive (DBP 70–105 and SBP 155–220 mmHg) (43). It has been suggested that the relationship between lower BP early after stroke and mortality is in part explained by the association with early cardiac adverse events reflecting LV dysfunction (110). Nevertheless, the prevalence of systolic dysfunction, the association with poor outcome, and the effect on cerebral perfusion suggests that avoiding moderate to high intensity aerobic exercise until recovery is recommended.

##### Time of onset and recovery of systolic dysfunction

LV dysfunction can develop after 1–4 days and can persist for more than 8 days post-stroke (described in detail in Supplementary Materials, Figure 1) (143, 150). Routine echocardiography is not typically recommended for the early management of acute stroke (15, 120, 151), except among patients with suspected embolic stroke despite normal neurovascular imaging (151). Cardiac-specific troponin and ECG are routine, however, and can provide insight on echocardiogram abnormalities (152–155).

#### Cardiac Arrhythmias

Cardiac arrhythmias are frequent in acute stroke and associated with higher morbidity and mortality (156). Up to 90% of patients will have ECG changes within the first 24 h of ischemic stroke and 22% are reported to have a cardiac arrhythmia; this is a common cause of death after acute ischemic stroke (157, 158). ECG abnormalities are more frequent in patients with SAH ranging from a prevalence of 27–100% with ~37.5% experiencing cardiac arrhythmias (144, 145, 157). Arrhythmias can be related to underlying cardiac disease, the stroke event itself, or simply coincidental. Cardiac arrhythmias are not only potentially life threatening (156), but like systolic cardiac dysfunction may compromise cardiac output and thus also have the potential to affect CBF. For example, atrial fibrillation can reduce cardiac output by as much as 17% in the non-stroke population (159, 160), limiting LV filling (“atrial kick”), and by extension, cerebral perfusion during exercise (161, 162). Ventricular arrhythmias, such as frequent premature beats, interpolated premature beats, bigeminy, and trigeminy can cause variable effects on hemodynamics including reduced ejection fraction and stroke volume (163) in people with no known history of stroke. The effect of the above arrhythmias on reduced CBF early post-stroke when CA is impaired has not been reported but is likely to be intensified.

##### Recovery and correlates of arrhythmias

The risk of clinically significant cardiac arrhythmias is highest in the first 24–48 h following stroke (see Figure 1 and Supplementary Materials) (164, 165).

Patients at higher risk of a clinically relevant arrhythmia following stroke are those who are older, those with more severe neurological deficits (NIHSS), and those with a greater lesion size (164, 165). Insular cortex ischemic strokes are associated with ventricular tachycardia/fibrillation, heart blocks, bradycardia, supraventricular tachycardia, and atrial flutter/fibrillation (156). Patients who fit this profile may benefit from more intensive cardiac monitoring strategies, such as ECG monitoring during the first exercise session or undergoing a pre-participation exercise stress test with ECG monitoring.

#### Myocardial Injury and Stress

Injury to the myocardium can occur in the acute stage of ischemic, SAH, and ICH in the absence of any cardiac cause. Cardiac lesions may not always be indicative of perfusion abnormalities (166) or affect cardiac output, but they have been characterized as subendocardial microinfarcts with possible damage to both myocytes and nerve terminals (129, 167). Van der Bilt et al. examined myocardium in 25 patients who died of SAH and 18 controls (131). Results revealed a significantly higher influx of inflammatory cells in the myocardium of SAH patients, indicative of myocarditis, relative to controls. Thrombi in intramyocardial arteries were found in 22 SAH patients and 1 control. Myocytolysis was detected in six SAH patients but not in controls.

Cardiac damage can also be detected by elevated serum cardiac troponin levels; a biochemical marker emanating from damaged sarcomeres. Assessment of troponin levels (subunits I and T) provides a high tissue specificity and clinical sensitivity for detecting myocardial necrosis (168). Elevated troponin levels have been detected in up to 21% of ischemic, 18% of ICH, and 52% of SAH strokes in people hospitalized with and without known cardiac disease (152, 155, 169–171). It is thought that elevated troponin levels may be due in part to a catecholamine-related contraction band necrosis (stunned myocardium) rather than underlying CAD (153, 172).

Elevated troponin is independently associated with higher in-hospital mortality, increased risk of delayed cerebral ischemia, and poor outcome across all stroke types (152, 169, 170, 173). In SAH, troponin level is positively correlated with stroke severity, arrhythmias, and regional wall motion abnormalities (153, 174, 175). Elevated troponin in people following ischemic stroke is also correlated with wall motion abnormalities (154). Specifically, of 137 consecutive hospital admissions for ischemic stroke, 17.5% (*n* = 24) had elevated troponin and 67% (*n* = 16 of 24) of those with elevated troponin had a new wall motion abnormality on echocardiogram. Wrigley et al. reported that, among >1,500 patients with acute ischemic stroke, 21% had elevated levels of troponin and 10% had echocardiogram findings of interest; most being reduced ejection fraction and wall motion abnormalities (155). Moreover, high troponin levels were independently associated with echocardiogram abnormalities. Most, but not all people post-stroke with elevated troponin will have concomitant ECG changes suggestive of myocardial ischemia (152, 153). Therefore, although echocardiogram results may not be available to detect cardiac manifestations post-stroke, both cardiac-specific troponin and ECG are recommended in acute stroke (15, 120).

##### Correlates of risk of myocardial injury

Cerebral infarctions involving specific brain regions including the insular cortex and right inferior parietal lobule have been associated with elevated troponin levels indicative of myocardial damage (176). Specifically, in patients with right middle cerebral artery infarction, damage to the insular cortex was involved in 88% of patients with elevated cardiac troponin and 33% of patients without elevated troponin levels in the weeks after ischemic stroke (176). Indeed, insular cortex and parietal lobe infarctions have been associated with adverse cardiac outcomes and cardiac dysfunction in human and animal model studies (177–179). In addition, cardiac troponin levels have been reported to be higher in patients with more severe strokes compared to those with less severe strokes (NIHSS) (180, 181) and positively associated with the stroke lesion volume (182).

##### Time of onset and recovery of myocardial injury

Kolin and Norris report that focal myocardial damage required at least 6 h to develop after onset of the acute neurological event and was not observed after the second week (183). Serial measures of troponin I in SAH reveal that troponin levels peaked between day 1–3 post-stroke and subsequently declined over 7 days (147, 153, 184, 185) (see Figure 1 and Supplemental Materials for more details). While the effects of exercise on the myocardium in the early stage of stroke in people with elevated troponin levels is not known, it may be prudent to maintain light aerobic activity for at least 7 days and up to 1 month post-stroke (see Figure 1) given the demonstrated microscopic damage and associated wall motion abnormalities.

#### Coronary Artery Disease

Myocardial infarction and cardiac surgery will not be reviewed because exercise guidelines following these events are well-documented (186). It is important, however, to note that coronary artery disease (CAD) can remain undiagnosed due to lack of symptoms and/or unremarkable resting ECG (187, 188).

## Mobilization and aerobic exercise in the hyper-acute and acute phases post-stroke

### Effect of Mobilization in Hyper-Acute to Acute Phases Post-stroke

A meta-analysis of nine randomized controlled studies (2,803 participants) implementing very early mobilization—defined as out of bed activity 24–48 h post-stroke—was published in 2017 (189). The AVERT study was the largest study in the analysis (20). Pooled analyses revealed that when compared to usual care control, early mobilization resulted in similar safety outcomes (e.g., falls with injury, neurological deterioration, death) but was not associated with additional functional improvements or mortality advantage at follow-up, or in reducing pulmonary infection, deep vein thrombosis, urinary tract infection, pulmonary embolism. One study in the meta-analysis, Sundseth et al. (190) randomized stroke patients post-stroke to early mobilization either within 24 h (*n* = 27) or 24–48 h (*n* = 29) after admission. The type and amount of early mobilization activity were not controlled: e.g., each patient was mobilized out of bed “several times per day.” The safety-related exclusion criteria included a mRS score ≤ 1 and acute coronary disease. No resting BP criteria or exclusion of people with orthostatic hypotension were reported. Results revealed non-significant trends for poorer outcome (mRS 3–6), higher death rate and dependency, and poorer neurological functioning in the very early mobilization group, although this study may be limited by the sample size.

In a subsequent study, 104 people with severe stroke were randomized to soft physiotherapy (20 min per day) vs. intensive physiotherapy (soft physiotherapy plus 45 min of intensive exercise/day) initiated within the first 72 h after stroke for 2 weeks (10 sessions) (191). Similar to the meta-analysis discussed above, no between-group differences were reported in mRS score, Functional Independence Measurement, mobility, change in Postural Assessment Scale for Stroke, or quality-of-life measure after 90 days. Unfortunately, no measure of “dose” of activity or pre-participation screening criteria based on resting BP, eye conditions (e.g., retinopathy), orthostatic hypotension, glycemic control, or cardiac abnormalities were reported, despite 70% of participants having a history of hypertension, 19% with diabetes, and 10% with cardiac issues.

The results of the most influential study in the 2017 meta-analysis also demonstrated a neutral and potentially deleterious effect of very early mobilization initiated within the first 24 h of stroke (20). The AVERT trial was a multi-center, single-blind randomized control trial conducted in 56 stroke units, 5 countries, and 2,104 ischemic and ICH stroke patients. The activity intervention was modest and included 10–30 min of active sitting, and/or a minimum of 10 min of standing, and/or walking that continued for 14 days or until discharge. The time to first mobilization for intervention and control was a median [interquartile range (IQR)] of 18.5 h (12.8–22.3) vs. 22.4 h (16.5–29.3), respectively. The median (IQR) time out of bed for intervention and control groups was 31 (16.5–50.5) vs. 10 (0–18) min per day, respectively. Three months post-stroke, a smaller proportion of people in the early mobilization group scored favorably (0–2) on the mRS compared to usual care (46 vs. 50%, respectively; adjusted odds ratio 0.73, 95% CI 0.59–0.90, *p* = 0.004). In particular, patients with severe stroke (NIHSS > 16, *n* = 291) and ICH (*n* = 255) tended to show a less favorable outcomes in the early intervention treatment, with ICH patients possibly more susceptible to death.

To further define a “dose” of out-of-bed activity associated with better outcomes regression models (two for usual care and two for all patients regardless of group assignment) [Table e-1 (192)] controlled for age, stroke severity (NIHSS), and frequency and duration of mobilization (either daily amount or total amount). An earlier start to mobilization and more frequent daily activity was a predictor of improved mRS outcome in all models. The only difference between the analyses was that in the usual care group, while daily amount of activity did not significantly influence outcome, a greater total amount of activity predicted worse outcomes. In both groups, greater daily amount and total amount of activity predicted poorer outcomes. This suggests that when mobilization is started later as suggested in the contemporary guidelines, greater amount of daily activity may not have an influence, but should be undertaken more frequently. The finding that a greater total amount of activity during hospitalization (up to 14 days) had a negative effect was likely influenced in part by longer hospital stay in those with greater medical complications but requires verification. Also, the variability in frequency and daily amount of out of bed activity may be in part driven by patient, family, institutional, health care professional, medical, and other factors. Thus, data from this secondary analysis should be viewed with caution.

As mentioned previously, most of the contemporary stroke care guidelines and position papers published since the release of the AVERT results advocate against “high-dose” or “intensive,” out-of-bed activities within 24 h of stroke onset without further specification of dose (15–19). Indeed, the dose of activity in the first 24 h was not reported in the AVERT study and although the median dose was reported as 31 (16.5–50.5) min of out-of-bed activity over ~14 days, it is likely that the first few mobilization sessions performed would be low intensity activity of shorter duration and then gradually progressed over the ~14 day intervention. While the AVERT investigators caution against interpretation (192), the results of Classification and Regression Tree (CART) analysis suggest that overall, younger individuals are likely to fair well. Older adults (76–86 years), without mild or severe strokes, have better outcomes with a median dose of ~2 sessions of 6.5 min of activity per day or, for longer duration of activity, a dose equivalent to at least 1–2 min of out-of-bed activity every hour of the day (i.e., ~11 sessions) if targeting the median dose. The optimal timing of the initial dose is not clear and it is possible that adverse outcome may be related in part to the type of initial activity prescribed, such as prolonged standing as discussed in section Protecting the Brain During Mobilization.

### Safety Screening Criteria for Very Early Mobilization in AVERT

Another consideration for improving outcomes with an early mobilization intervention is related to having appropriate pre-participation screening criteria. The AVERT study excluded participants with a resting SBP of <110 or >220 mmHg. In acute stroke, where CA is disturbed and many have a history of hypertension and risk for orthostatic hypotension, protection would likely be compromised in some patients using this criterion. In addition, pre-participation criteria for a safe lower limit of resting BP should be re-evaluated (see section Peripheral and Cerebral Circulatory Considerations for Exercise and Mobilization Guideline 1.0). Another factor to consider is that almost one-quarter of the patients in the AVERT study had a diagnosis of diabetes and screening criteria for hyperglycemia was not reported. Further, one-quarter of the patients from both groups in the AVERT study had a diagnosis of atrial fibrillation, although it is unclear how many were currently in this rhythm, should have followed Guidelines 3.0–5.0, Tables 4–6 to avoid cerebral hypoperfusion episodes associated with activity until recovery of CA.

**Table 4:** Guideline 3.0: Precautions for avoding orthostatic hypotension.

OH is common post-stroke. There is an opportunity to use this clinical indication to guide exercise and early mobilization. While there is some evidence that repeated episodes of standing may improve orthostatic tolerance over time in some populations (54), until there is further research specific to stroke, precautions to prevent OH should be taken until at least after the expected recovery of CA when the brain is better protected from hypotensive episodes. In view of the high prevalence of OH in the early phases of stroke that may be asymptomatic and result in reduced CBF, the following precautions and strategies are suggested: Factors Predisposing People to OH Requiring Careful Monitoring ◦ Patients who experienced any of the following 12 symptoms of orthostatic intolerance pre-stroke (symptoms would present within 3 min of standing and resolve when sitting or lying down): dizziness, lightheadedness, fatigue, blackouts, nausea, instability, ringing in the ears, vertigo, headache, syncope, confusion, and sweating. ◦ People with tightly controlled blood pressure (e.g., SBP below 120 mmHg and/or DBP below 80 mmHg in ischemic stroke) (55). Refer to Guideline 1.0 for lower blood pressure limits prior to commencing early mobilization/exercise prescription. ◦ People with diabetes, dehydration (blood electrolytes, urea nitrogen, and creatinine), anemia (hemoglobin and hematocrit levels), hemicraniectomy, and intracranial atherosclerotic stenosis. ◦ Medications such as beta-adrenergic blockers, renin-angiotensin system antagonists, diuretics, antidepressants, or sedatives, which can cause or aggravate OH (56). Avoid exercise after large meals (57). In those with signs and/or symptoms of OH, schedule exercise or mobilization for those prescribed beta blockade medication at a time of day when the medication is less effective unless the risk of high blood pressure outweighs risk of hypotensive episode. Minimize posture change or institute an incremental change in backrest tilt posture from 30–50 to 70 degrees (>10 min for each increment) concomitant with lower limb movement (active or passive) to activate the muscle pump, when possible (58). Timing of Assessment for OH: Measure changes in blood pressure and heart rate and monitor symptoms when moving from supine (10 min supine) to standing (after 1 and 3 min) at the same time of day as the mobilization or exercise session will be performed (59). Until further research has been conducted, we suggest the following OH thresholds based on resting blood pressure (systolic/diastolic; SBP/DBP) values: ◦^*^SBP < 128 and/or DBP < 82: A sustained reduction in either SBP or DBP of at least 15 or 7 mmHg, respectively, after 3 min of standing or after a head-up tilt of at least 60 degrees with or without OH symptoms (60). ◦ SBP 128–158 mmHg and/or DBP 82–102 mmHg: A sustained reduction in either SBP or DBP of at least 20 mmHg or 10 mmHg, respectively, after 3 min of standing or after a head-up tilt to at least 60 degrees with or without OH symptoms. ◦ SBP > 158 mmHg and/or DBP > 102 mmHg: A sustained reduction in SBP and/or DBP of at least 30 or 10 mmHg, respectively, after 3 min of standing or after a head-up tilt to at least 60 degrees with or without OH symptoms. Using an ambulatory blood pressure monitoring device, measure blood pressure for the first 2–3 training/mobilization sessions. Monitor from supine through to 90 min post-exercise. Repeat monitoring when there is a change in exercise modality or change in medication (listed above) in those with suspected OH (i.e., measured or in people with symptoms of orthostatic intolerance listed above). Precautions should continue to be practiced throughout care in those with signs and/or symptoms of OH as it is likely to continue into chronic stroke especially in those with coexisting diabetes.	

* *This conservative recommendation is based on data demonstrating subclinical OH is associated with increased risk of dementia (60), most people post-stroke are asymptomatic during OH (61) and people post-ischemic stroke with SBP ≤ 127 mmHg and/or DBP of ≤82 mmHg are more susceptible to OH (55)*.

**Table 5:** Guideline 4.0: Precautions for preventing adverse effects from prolonged standing.

Avoid prolonged (> 5 min) stationary standing, especially after prolonged sitting. ◦ It is possible that even shorter periods of prolonged stationary standing may be detrimental. ◦ In a situation that necessitates prolonged standing, recommendations are to engage the muscle pump by doing ~10–15 s (4–5 repetitions) of rhythmic heel raises or squats (with support if required) alternating with 60 s rest. Early mobilization strategies for those with significant hemiparesis and/or poor lower extremity motor control is to do the following: ◦ Replace placid standing with side-to-side or forward and backward stepping (support by non-affected upper extremity) that would force at least passive movement of the ankle joint. ◦ Perform passive or active ankle movements on a BOSU ball with affected leg in standing with support by non-affected upper and lower extremity. Activate the non-affected limb by also performing heel raises.	

**Table 6:** Guideline 5.0: Precautions to prevent post-exercise or mobilization hypotension.

Strategies to counteract post-exercise hypotension should be practiced in the early phases post-stroke in the setting of compromised CA. While the lasting effects of post-exercise hypotension (PEH) are not known, these precautions are not likely to significantly alter benefit, be a burden to the patient, or increase risk.It should be emphasized that people who experience post-exercise symptoms or syncope should be investigated for other serious pathologies including arrhythmias, carotid disease, cerebral vasospasm or other issues. Pre-exercise precautions Avoid exercise in the early morning as CA is more likely to be impaired (62, 63). Avoid exercise in the hot and/or humid weather. Exercise performed with additional heat stress may worsen the degree of orthostatic intolerance and extend the deficit in CA even after return to resting body temperature in healthy individuals (64). Ensure adequate hydration prior to and during exercise and replace fluids post-exercise (64). Avoid a large carbohydrate meal and allow at least 2 h post-meal before initiating exercise to reduce postprandial splanchnic hyperemia and subsequent hypotension. When designing an exercise and risk factor modification program, the education component should be delivered prior to exercise, so as to avoid static upright postures (e.g., prolonged sitting) post-exercise. Exercise precautions In at least the acute phase post-stroke, light intensity exercise is recommended while avoiding high intensity exercise owing to increased risk of PEH. This is especially important in those who have experienced symptoms post-exercise and those with resting hypertension or borderline hypertension. ◦ Symptoms of PEH can include dizziness, nausea, faintness, visual disturbances, hearing disturbances, and fatigue Shorter exercise protocols have been shown to elicit less of a PEH response than longer protocols. Therefore, exercise intervals of 5–10 min each, alternating with active recovery periods should be prescribed. Active recovery includes seated/standing activity that engages the skeletal muscle pump. In some cases, support stockings/socks may be of benefit (65, 66). Post-exercise precautions The cool-down period should not be neglected and should be a formal component of the exercise prescription. ◦ The cool down period should include ≥5 min of a gradual ramping down to very low intensity activity. A rapid decrease in blood pressure post-exercise results in less effective dynamic CA especially in the first 10 min of recovery post-exercise (67). ◦ People with resting hypertension or borderline hypertension should include a 10-min cool down period as PEH is greater in magnitude and can last longer and may be further exacerbated when ambient temperatures and humidity exists. ◦ On the stationary cycle, gradually reducing cycling resistance should be the primary way to reduce workload in the cool-down period while maintaining pedaling cadence to allow more frequent muscle pump activity. Repeated rhythmic ¼ squats or heel raises should be performed for at least 10 min and up to 30 min following cessation of exercise. This should be sufficient to move blood toward the heart. ◦ After cool-down, lower limb movement should be periodically undertaken for at least 10 min following cessation of exercise to engage the mechanical muscle pump and reverse the shift of blood volume as this has been shown to reduce occurrence of PEH in healthy individuals (65). ◦ Engage the muscle pump by doing ~10–15 s (3–4 repetitions) of rhythmic ¼ squats or heel raises (with support if required) alternating with 60 s of rest for at least the first 10 min post-exercise (~25 in total). These should then be repeated 2 more times (every 10 min for a further 20 min). ◦ Strategies for those with severe hemiparesis and/or poor lower extremity motor control is to perform passive lower limb movement (68) or perform side-to-side or forward and backward stepping (support by non-affected upper extremity). Affected-side ankle movements on a BOSU ball or other activities such as seated heel raises can also be performed.	

### Effects of Aerobic Exercise Within 48 H Post-stroke

To our knowledge there is only one study examining aerobic training within 2 days of stroke onset. Strømmen et al. conducted a single group prospective study in 20 people with mild to no disability (mRS of 0–2) that initiated exercise 41.5 ± 14 h after onset of symptoms (193). The intervention included two sessions of low intensity (50% of predicted heart rate reserve; HRR) treadmill training (body weight support when needed) per day for the first 5 days and two sessions 30 days later (193). Each session was 30 min in duration, with rest breaks (sitting or standing) as needed. Exclusion criteria included symptoms, infection, unstable cardiac condition, resting SBP above 180 mmHg, and conditions hindering treadmill training. Of the 20 participants, over half developed non-serious adverse events occurring in 14% of all 224 treadmill training sessions. Specifically, eight people developed 19 episodes of dizziness (with two patients ending four sessions pre-maturely due to dizziness), three people developed blisters or superficial wounds, one person had three non-injurious falls getting on or off the treadmill, one patient had five episodes of pain in the lower extremities, and one patient had three episodes of tiredness. Not included in the adverse events, nine patients became exhausted and ended a total of 24 exercise sessions early. No neurological deterioration was detected. Participants attained the target exercise intensity in only 31% of sessions. The difficulties encountered by these minimally disabled patients attempting to reach an exercise intensity slightly above “light” suggests that our recommendation to initiate light intensity exercise in the acute phase of exercise is a more feasible and realistic goal for patients. Counteracting adverse events is discussed in section Protecting the Brain During Mobilization. We await the results of ongoing clinical trials examining early exercise interventions (194).

The preclinical data suggest that very early exercise (i.e., within 6 h) may exacerbate brain injury, while early (i.e., ~24 h) and relatively late training (i.e., >3 days) may be beneficial. The Supplemental Materials provides further description on relevant animal studies but the preclinical field of research is outside the scope of this review.

## Protecting the brain during mobilization

### Orthostatic Hypotension (See Table 4 Guideline 3)

Orthostatic hypotension (OH) can impact stroke survivors. OH is defined as a sustained reduction in either SBP of at least 20 mmHg or DBP of at least 10 mmHg, within 2–3 min of standing, or after a head-up tilt to at least 60 degrees, preceded by a 10-min period of quiet lying (195). For resting supine SBP of >160 mmHg, the OH threshold for a drop in SBP is increased to 30 mmHg. Symptoms of OH can include dizziness, nausea, dyspnea, diaphoresis, and diplopia that can sometimes lead to vasovagal syncope (196, 197). The pathogenesis of OH helps to elucidate the possible mechanism for adverse long-term outcomes. When assuming an upright posture, blood volume is redistributed below the diaphragm (198). This leads to a decrease in venous return, cardiac output, and arterial BP. In healthy individuals, a compensatory reflex is activated by baroreceptors in the carotid arteries and aorta to restore BP and cardiac output by increasing heart rate, contractility and vascular resistance. In people following stroke and the elderly, however, arterial stiffening likely impairs cardiovagal baroreflex sensitivity (199, 200) and interferes with these countermeasures. CA dysfunction likely intensifies the effect. Moreover, primary baroreflex dysregulation has been identified as a cause of OH (201).

#### Prevalence and Incidence of Orthostatic Hypotension and Hypotensive Episodes in People Post-stroke

Among 71 stroke adults in in-patient rehabilitation, 52% had OH during a tilt table test measured within 3 days of stroke (61). It is notable that 68% of these cases were asymptomatic, emphasizing the importance of careful BP monitoring during early phase mobilization post-stroke. Further, Carlsson et al. reported that 23% of 226 patients within 4 weeks of mixed diagnosis stroke demonstrated OH, which persisted for up to 1 year (202). In a small study of the early phases post-ischemic stroke (*n* = 13), Treger et al. reported that 40% of individuals exhibited symptomatic OH at 1 week of in-patient rehabilitation (range 15–45 days post-stroke). One month later (45–75 days post-stroke), these patients had the same symptoms, albeit less severe in some cases (203). Panayiotou et al. reported a slightly lower incidence of postural hypotension (19% of 40 people) 1–2 days following acute mild or moderate ischemic stroke. This study reported hypotension after 1 min of standing but pressure had recovered after 5 min in most of the patients (204). This study also provides preliminary evidence that OH prevalence may be related to stroke severity.

Langhorne et al. monitored BP (using either automatic continuous or manual methods) in patients randomized to early mobilization vs. standard care (205). Among 32 patients post-stroke in the first 72 h of admission, there were 28 episodes of DBP dropping below 70 mmHg and 5 episodes where it rose above 120 mmHg, 18 episodes of SBP dropping below 110 mm Hg and 2 where it exceeded 220 mmHg, 7 episodes of bradycardia (heart rate dropping below 50 bpm) and 15 episodes of tachycardia (heart rate exceeding 100 bpm). No differences in the frequency of these events by early mobilization verses usual care were reported; however, an unfavorable neurological impact may be greater in the early mobilization group given BBB and CA dysfunction. Unfortunately, the events that may have precipitated these episodes such as posture change, activity, prolonged standing, post-exercise hypotension were not reported but serve to highlight the frequency of these events that occur at a time when the brain is vulnerable to hypoperfusion and hyperperfusion.

#### Orthostatic Hypotension and Activation of the Coagulation Cascade

Physical countermeasures and other strategies may mitigate the effects of OH for stroke survivors, but this concept is largely untested. Furthermore, avoiding activity at times when OH is probable may contribute to better long-term outcomes. We discuss some possible mechanisms. First, changes in posture may trigger the coagulation cascade. In an observational study of 178 adults with unexplained syncope (non-stroke), activation of the coagulation cascade occurred after only 3 min of head up tilt at 70 degrees (206, 207). This hypercoagulable state can persist for ~20 min following a postural change (208). These changes were observed in both individuals with OH as well as those with other syncope etiology. Orthostatic-driven coagulation may in part explain the increased risk of cardiovascular events that are reported in people who experience OH.

#### Orthostatic Hypotension and Reduced Cerebral Blood Flow Velocity

In addition to the hypercoagulable state, repeated acute hypotensive episodes early-post-stroke may contribute to hypoperfusion. Pooled data from four studies demonstrated a significant decrease in CBF velocity when head position moved from either 0 or 15 degrees to a 30-degree upright head position (209). Patients (*n* = 57) were within 6 days of mostly large vessel ischemic strokes. One study in the review measured the impact of a change in backrest tilt following large ischemic stroke with 7 of 18 participants having had decompressive hemicraniectomy (210). Moving from horizontal to 15 degrees and then to 30 degrees over a two step 10-min period decreased CBF velocity by 25% and also reduced intracranial pressure and cerebral perfusion pressure. BP showed a significant decline from baseline at both 15 and 30 degrees. The decrease in CBF was even larger in the subset of patients with hemicraniectomy. The rate of posture change that would minimize hypoperfusion and the time course for CBF and BP to return to baseline levels after posture change is unknown and therefore an area of future investigation. Such information would help define specific guidelines for protecting individuals from repeated episodes of hypoperfusion and increased fall risk.

The coexistence of diabetes may increase the prevalence of OH in patients with stroke and may intensify the effect on CBF, as the prevalence of OH in the pre-diabetic and diabetic population is ~18 and 26%, respectively (211). One study documented a reduction in mean CBF velocity of 23% upon active standing from a supine position in people with diabetes and no stroke (116). The prevalence of OH and effect on CBF in people with both diabetes and stroke requires investigation.

#### Orthostatic Hypotension Is Associated With Cognitive Decline and Poorer Physical Function

Physical and cognitive functions are both relevant in the context of OH. The effect of both hypotensive episodes and aortic stiffness on cerebral function has been measured in cross-sectional and longitudinal studies, but not in people with stroke. These studies demonstrate that there is an association between OH and cognitive decline (60, 212). Indeed, the impact of OH on cognitive decline is significant, with a pooled analysis of data indicating a 21% (95% confidence interval: 9–35%) increased risk of dementia (60). Of note, subclinical OH (i.e., a fall of ≥15 mmHg in systolic and/or ≥7 mmHg in DBP after 2 min of standing from sitting) with symptoms in the previous week also increased the risk/incidence of cognitive impairment in older hypertensive individuals (60).

Overall, these data indirectly support the notion of careful monitoring and prevention of OH episodes during mobilization for people post-stroke be considered. There are clear research opportunities, including randomized trial design, that build from the limited literature (213). As little as a 15-degree change in head position is shown to decrease CBF significantly in the acute phase post-stroke. Thus, minimal and gradual changes in head and body position preferably with concomitant stepping or lower limb movement to activate the venous muscle pump to counteract pooling of blood (58), should be carried out carefully when preceding initiation of aerobic exercise or mobilization.

#### Orthostatic Hypotension and Increased Falls Risk

OH is clinically important after stroke because of the increased fall risk. Although the incidence varies among studies, up to 37% of post-stroke inpatients report at least 1 fall (214–216), accounting for up to 40% of all adverse hospital events post-stroke (217). Surprisingly few studies, if any, have prospectively examined the association between falls and OH early post-stroke. This may explain why current risk prediction models have had unacceptable performance in predicting falls post-stroke (218).

#### Rethinking the Definition of OH for Detecting Clinically Relevant OH Post-stroke

Regarding detection of clinically relevant OH, there is no empirical evidence to support that the established BP decline thresholds defined as OH will provide cerebral protection in early stroke. It is possible that in the presence of impaired CA, a less dramatic fall that does not exceed these thresholds could be of equal clinical importance in both those with and without hypertension. A re-evaluation of this threshold is needed as hypoperfusion during the hyper-acute and acute phases post-stroke may result in a collapse of the blood supply to the vulnerable ischemic penumbra leading to stroke progression. Indeed, the safety related criteria for excluding patients from participating in the AVERT study was if the patients' SBP dropped by more than 30 mmHg when the back of the bed was raised to >70° of hip flexion or during sitting both for normotensive and hypertensive individuals (20). This may have in part explained the less favorable outcomes by 3 months in the early vs. late mobilization cohort, especially in those with more severe stroke (NIHSS > 16, *n* = 291), a cohort that may have greater CA impairment. Future studies should determine the BP reduction threshold that results in significant reductions in CBF velocity in normotensive and hypertensive individuals with and without impaired CA early post-stroke to inform safety related screening criteria. A more conservative guideline for reduction in BP upon standing should be considered when mobilizing and prescribing exercise until further research is conducted in this area.

### Prolonged Standing (See Table 5 Guideline 4.0)

Prolonged static standing (i.e., >5 min) is an orthostatic and CA challenge; it is an activity with no dynamic movement and can lead to a reduction in arterial BP and cardiac output. As in OH, prolonged standing can trigger the coagulation cascade, called orthostatic hypercoagulability. For example, when healthy individuals stand stationary for ~20–30 min, venous pooling of ~20% of the blood volume occurs in the lower extremities with a subsequent plasma volume loss to surrounding tissue of ~12% (219, 220). This orthostatic stress and plasma shift of filterable elements and water into the interstitial space is associated with an increased concentration of coagulation factors and other proteins that are larger and non-diffusible in the lower extremity vasculature, subsequently causing hypercoagulability (208, 220, 221).

A recent study measured coagulability in 22 patients within 1 year of mild ischemic stroke (most were prescribed antiplatelet medication) and 22 age-matched healthy controls before and after 5 min of sitting followed by 6 min of quiet prolonged standing (221). The orthostatic challenge resulted in a significant activation of the coagulation system in both groups. However, activation was more easily shifted toward a higher hypercoagulable state in ischemic stroke than in healthy controls. This study demonstrates that a mere 6 min of inactive standing can be problematic post-stroke. A decrease in plasma volume, an increase in plasma protein, and a net higher coagulability has been demonstrated in healthy subjects after 30 min of standing (220). Other types of prolonged inactivity (recumbency and sitting) have also been shown to activate the coagulation cascade (222).

Prolonged standing leads to reduced venous return, cardiac output, and BP. When this is not countered by a baroreflex mediated increase in sympathetic outflow and vagal inhibition, the reduced cardiac output may threaten brain perfusion (223). CBF, in part, depends on cardiac output (139). Heel raises are a simple strategy to counter these effects and activate the skeletal muscle pump. Increasing intravenous pressure facilitates venous return to the heart. Faghri et al. demonstrated that 30 min of stationary standing by 15 able bodied and 14 spinal cord-injured subjects resulted in significant reductions in cardiac output in both groups (224). During 30 min of dynamic standing, however, both groups were able to maintain cardiac output at baseline levels by way of either electrical stimulation (in spinal cord injury) or voluntary activation (in controls) of postural leg muscles (10–15 s of heel raises with 60 s rest repeated for 30 min).

In people with lower extremity hemiparesis, an inability to voluntarily activate the muscle pump optimally may intensify impaired venous return and the subsequent effects. Passive dorsi-flexion and ankle rotation can increase mean and peak blood velocities in the common femoral vein in healthy individuals (68). Therefore, an early mobilization strategy for those with significant hemiparesis and/or poor lower extremity motor control is to replace placid standing with side-to-side or forward and backward stepping (support by non-affected upper extremity) that would force at least passive movement of the ankle joint.

Unfortunately, studies examining in-hospital activity tend to cluster as opposed to distinguish between standing, walking, and upright forms of activity (11, 20). This was the case in the AVERT study; thus, there may be scientific justification to isolate prolonged standing from other forms of mobilization in future trial design (20). Two of the three types of mobilization activities prescribed in the AVERT study were standing (i.e., a minimum of 10 min of standing and/or walking) and sitting. The results from the CART analysis of the AVERT study, indicating a benefit from activity intervals shorter than 6 min, aligns with research presented in this section which shows that the coagulation cascade is triggered after only 6 min of prolonged standing and only 3 min following a change in posture.

Also, shorter exercise protocols may elicit a smaller post-exercise hypotensive response than longer protocols (225, 226). Although the results are mixed, CA has been reported in some studies as being more impaired in those with more severe strokes. This may in part explain why those with more severe stroke had a more favorable outcome with more frequent sessions compared to less daily sessions in the AVERT study. Specifically, CART analysis revealed a more favorable outcome (mRS of 0–2) in patients with more severe stroke (NIHSS of >13.5) who performed a median of >2.75 daily sessions (16.2%) rather than less daily sessions (3.7%), and a more favorable outcome for those in the usual care group than the intervention group, potentially due to the later initiation of mobilization. This more severe cohort might also have greater mobility deficits and be more likely to be prescribed static standing or sitting out-of-bed activities rather than walking. Thus, more frequent instead of longer daily sessions may be of some benefit. Indeed, it is likely that upon first mobilization within the first 24 h, most of the activity in people with more severe motor impairments would be sitting or standing and gradually progressed over the 14 days of the intervention to walking, placing many at risk. Therefore, until further investigation, delaying prolonged static standing, especially in those with severe stroke or instituting countermeasures is recommended until there is some recovery of BBB and CA. Future studies should test these hypotheses and examine the effects of walking, standing, or sitting separately to help determine safe prescription parameters.

## Protecting the brain after and during aerobic exercise

### Post-exercise Hypotension (See Table 6 Guideline 5)

Post-exercise hypotension (PEH) is a reduction in arterial BP below resting levels that lasts minutes to hours following a bout of dynamic exercise, with a nadir typically at ~10–30 min post-exercise (227–229). During exercise, BP and cardiac output increase; after cessation of exercise, however, the average decline in BP can be ~8/9 (SBP/DBP) mmHg below baseline in non-stroke populations [reviewed by MacDonald et al. (230)]. The reduction in BP can be large enough to lead to presyncopal signs and symptoms, and possibly syncope (231, 232). The causes of PEH remain unclear but may result from peripheral vasodilation that is not completely offset by a matched increase in cardiac output. Just as with OH and prolonged standing, reduced cardiac output during PEH may threaten perfusion to brain tissue (223) because CBF is dependent on cardiac output (139). Notably, PEH compromises CA function in healthy individuals (67, 93), so it likely has an exaggerated effect on cerebral hemodynamics in people with stroke who may already have compromised CA. Also, while there is considerable heterogeneity in the PEH response, it appears to be greater in magnitude and lasts longer in hypertensive compared to normotensive individuals (227, 232). The average reduction in SBP/DBP is ~14/9 mmHg in the hypertensive population (230). As hypertension is a common risk factor for stroke and is commonly elevated in the hyper-acute and acute phases post-stroke, an even more pronounced reduction in BP may occur. The prevalence and effects of PEH in people with stroke with or without hypertension, however, is an area that requires further research.

Subjective symptoms of pre-syncope that are associated with PEH are dizziness, nausea, faintness, visual disturbances, hearing disturbances, and fatigue. Previous studies have reported a high prevalence of symptoms similar to these mostly observed in the acute phases post-stroke. In section Mobilization and Aerobic Exercise in the Hyper-Acute and Acute Phases Post-stroke, we reviewed a study that demonstrated that over half of a group of 20 stroke patients undergoing an aerobic treadmill exercise intervention a mean of 42 h post-stroke developed non-serious adverse events, some of which included dizziness and tiredness (193). Further, Langhorne et al. reported that from among 32 patients with and without an early mobilization intervention stroke, there were 28 episodes where DBP dropped below 70 mmHg and 18 episodes where SBP dropped below 110 mmHg within 72 h of stroke onset (205). While the circumstances under which these symptoms and episodes of low BP arose were not reported, it is reasonable to assume that some were related to OH or PEH, particularly given the high prevalence reported in healthy individuals (233).

#### Aerobic Exercise Characteristics and Post-exercise Hypotension

An understanding of the exercise characteristics that may precipitate PEH will help to develop countermeasures for prevention. Exercise engaging a greater volume of muscle mass and longer compared to shorter exercise protocols promotes greater reductions in BP during the recovery period (225, 226). This may be another factor contributing to the pre-specified secondary finding of the AVERT study where better outcomes resulted with a greater frequency of daily mobilization sessions when total time remained constant. Even very low intensity exercise can reduce CBF below resting levels in the post-exercise recovery period. In one study, 11 healthy individuals were assessed using PET oxygen-15-labeled water following 20 min of mostly very low intensity exercise (30% of estimated HRR). Results revealed that regional CBF decreased 8–13% during PEH compared to rest (234).

A series of studies demonstrate that PEH is more pronounced with higher intensity exercise than lower intensity exercise resulting in accelerated development of PEH, greater impairment in post-exercise CA and reductions in CBF velocity (235). Indeed, more intense exercise results in impaired functionality of dynamic CA measured in the post-exercise recovery period in healthy individuals (67, 93, 236). In healthy sedentary individuals, Boeno et al. reported that high intensity interval training resulted in reductions in SBP of 18 mmHg that occurred at the 15th min of recovery while continuous training at 70% of maximal heart rate (matched for volume) resulted in a reduction of 13 mmHg at the 30th min, compared to resting measures (228). Mündel et al. measured post-exercise orthostatic tolerance during an orthostatic challenge (head-up tilt and lower body negative pressure) in eight young healthy volunteers following 1 h of cycling exercise at intensities of 30 and 70% of predicted HRR (229). Following exercise at 70% HRR, the time to presyncope occurred 32% sooner than following exercise at 30% HRR (15.9 vs. 23.6 min, respectively).

#### Preventing Post-exercise Hypotension and Reduced Cerebral Blood Flow Velocity

Syncope typically occurs when the person is standing motionless for the first 5–10 min post-exercise given the loss of the muscle pump to aid in venous return. Passive recovery by standing or sitting places the vasodilated vessels in the periphery below the heart level and may exacerbate venous pooling. Table 6 includes strategies to prevent or mitigate the effects of PEH.

It is important to point out that while we advocate the mitigation of significant PEH in the early phases post-stroke when CA is impaired, its summative effects over time may contribute significantly to the favorable reduction in BP which may be beneficial when CA is functional (late subacute and chronic phases depending on stroke type), thereby providing a potential cardiovascular benefit. Further research is required to confirm this in the stroke population.

### Elevation and Rapid Fluctuations in Mean Arterial Pressure Related to Aerobic Exercise (See Table 7 Guideline 6.0)

The American Heart and American Stroke Association guidelines recommend physical activity and exercise across all phases of stroke recovery (237). Our review of animal studies (see Supplementary Materials) suggests initiating exercise in the hyper-acute post-stroke phase with caution if at all. There is scant evidence from human studies to oppose this recommendation. The relationship between CBF and exercise-induced changes in cardiac output, BP, metabolism, arterial blood gases, and neurovascular innervation in healthy populations is poorly understood and far less is known about this complex set of associations in the stroke population. Until there is more research to elucidate the response in the early phases post-stroke a cautious approach should be taken. Given that exercise intensity is the most important parameter of the aerobic exercise prescription from a brain safety and overall efficacy point-of-view, the following section will provide temporal guidelines with respect to aerobic exercise intensity based on available evidence. Strategies will be provided to avoid catecholamine surges and excessive elevation and rapid fluctuations in BP within the first 3 months post-stroke until the expected restoration of CA, BBB, and cardiac function. For a summary of exercise prescription guidelines, see Table 7 Guideline 6 and the Figure 1.

**Table 7:** Guideline 6.0: Strategies to minimize catecholamine surge and increases in mean arterial pressure.

Aerobic exercise intensity (see Figure 1): Up to 1 month post-stroke: Mobilization and then gradual progression to light and then moderate intensity aerobic activity [~10–15% below the level of the anaerobic threshold (ATh)]. Increase light intensity total duration first by ~5–10 min every 1–2 weeks to ≥20 min (non-continuous preferred in 5–10 min intervals) then gradually increase intensity. The aim being to achieve moderate intensity exercise at the end of the 4 week period in higher functioning less medically complex patients. 1–3 months post- stroke: Gradual progression from moderate intensity (~10–15% below the level of the ATh) to the ATh if appropriate. First increase duration from 20 min to 30–60 min and then increase intensity. More than 3 months post-stroke: Gradual progression to greater than moderate (ATh) to high intensity continuous or interval aerobic training if appropriate (preferably based on results of a graded exercise stress test with ECG monitoring). Patients should be appropriately screened for participation and exercise strategies for safe prescription implemented. Exercise and mobilization should be prescribed on a case-by-case basis. Allow time for physiological adaptation after progression of an exercise parameter. In people with borderline high resting blood pressure in the first month post-stroke, prescribe exercise that engages a small amount of muscle mass. In those not at risk of OH and prescribed beta-blocker medication such as Metoprolol (Lopressor) and Atenolol (Tenormin) perform aerobic exercises at a time when the medication is at maximum effect (i.e., ~2–4 h after oral administration depending on dose). Avoid morning exercise in the early post-stroke phase until more research has been conducted in people following stroke.	
**Strategies to reduce mean arterial pressure fluctuations**	
Avoid exercise that results in rapid and large fluctuations in MAP such as rowing and high intensity interval training, until at least 3 months post-stroke. Avoid a sudden transition in exercise intensity by including a gradual ramping up or down in intensity during the warm up and cool down period. Avoid the Valsalva-like maneuver (breath holding) and avoid exercise with an isometric component (like rowing).	

#### Aerobic Exercise Intensity

Well-established evidence demonstrates that greater gains in cardiorespiratory fitness (CRF) are possible with higher intensity exercise in stroke and other populations (238–241). Given that increases in CRF are associated with reductions in cardiovascular event rates (242–245), and in view of the progressive nature of cardiovascular disease (246, 247) efforts to train individuals following stroke to optimal target intensity levels are warranted. Indeed, epidemiological and clinical evidence demonstrates CRF as a stronger predictor of mortality than smoking, hypertension, type II diabetes, and high cholesterol (243, 248–252). In addition to CRF benefit, there is compelling evidence that higher intensity aerobic exercise training in the early subacute to late subacute phases of stroke provides an advantage to mobility (253, 254) and to cognitive function in late subacute and chronic stroke and healthy populations (255–261). However, to our knowledge the acute and chronic effect of aerobic exercise intensity on resting and dynamic middle cerebral artery blood flow velocity has never been measured at any time following the stroke event. Indeed, the benefits observed from aerobic exercise on cognition and mobility has not been replicated in the hyper-acute to acute phases post-stroke. Currently, the American College of Sports Medicine exercise recommendations for people following stroke advocate prescribing moderate intensity cardiovascular exercise at 40–70% of HRR or an RPE of 11–14 (“light” to <“hard”) on the 6–20 Borg scale (186). These guidelines do not specify the timing of when to safely initiate or progress patients to higher intensity aerobic exercise.

#### Higher Intensity Training in the Early Phases Post-stroke

In view of the data presented in section Peripheral and Cerebral Circulatory Considerations for Exercise and Mobilization, exposure to higher intensity exercise (95) within 1 month of stroke may increase the risk of BBB mechanical breakdown and cerebral hyperperfusion injury (Figure 1). The risk could be intensified by elevated and dynamically changing resting BP and structural cardiac complications and arrhythmias. The ischemic penumbra is vulnerable during this time period, and there is a risk of stroke progression, hematoma expansion in ICH, and delayed ischemia and vasospasm in SAH. There is also evidence presented earlier that dynamic CA measured during exercise as well as in recovery in people without stroke is further impaired with high intensity exercise when compared to lower intensity exercise (67, 93, 229, 236, 262). Conversely, there may be benefit from chronic adaptations to early aerobic exercise that leads to improved CA or BBB function (263). However, this has not been tested in people in the early phases post-stroke nor is there evidence of an exercise intensity effect on improved neuroprotection.

Is there an exercise intensity threshold that is safe in the early phases post-stroke? Studies in healthy individuals show that during incremental aerobic exercise, CBF gradually increases in parallel with exercise intensity until ~60–70% of VO_2max_. Exceeding this intensity typically results in either a plateau or progressive reduction in CBF toward resting values as induced by cerebral vasoconstriction in concert with exercise-induced hyperventilation [reviewed in Smith et al. (264)]. Prescribing exercise at an intensity below the level of expected peak CBF velocity in the early phases post-stroke may be a safe target to prevent hyperperfusion given that impaired CA may not induce cerebral vasoconstriction at the critical intensity for cerebral protection.

The degree to which CBF and cerebral perfusion are affected during exercise is related to the magnitude of hypocapnia induced by hyperventilation and this can occur at a different intensity relative to VO_2max_ for each individual. Olson et al. conducted a study of 14 healthy individuals and reported that the reduction in CBF velocity occurred above the ventilatory anaerobic threshold (ATh) (at the nadir of V_E_/VCO_2_) (265). In a similar study of 14 healthy individuals, maximal CBF velocity occurred at the exercise intensity just below the ATh (respiratory exchange ratio ≤1.0) during graded incremental exercise tests performed to exhaustion (266). Therefore, there may be utility in using the ATh as a metabolically uniform and individualized threshold intensity for determining safe exercise early post-stroke (i.e., an intensity level that occurs prior to achieving peak CBF velocity). Although CBF response to exercise above and below the ATh under conditions of impaired CA has not been reported, since CBF velocity peaks at the ATh in healthy individuals, it may be prudent to prescribe exercise intensity below the ATh or where a non-linear increase in ventilation occurs. A non-linear increase in ventilation typically occurs at the level of the ATh, owing to excess CO_2_ production (267). Therefore, a graded cardiopulmonary exercise test that is terminated after achieving the ATh early post-stroke would help to determine the intensity parameter of the exercise prescription. However, investigation into the safety of such a test is required. Alternatively, another strategy for guiding exercise intensity is to use the Talk Test. The Talk Test can be used as a surrogate of the ATh (268, 269). The premise is that it is difficult to talk when exercising at or above the ATh.

While exercise just below the ATh may be a safe intensity threshold, it may be a challenge for most people to reach and sustain this intensity owing to disability, fatigue, poor balance, and deconditioning (270). In a study described in section Effects of Aerobic Exercise Within 48 h Post-stroke, people were challenged to reach less than moderate intensity effort when treadmill aerobic exercise was initiated 41.5 ± 14 h post-stroke (193). Over half experienced symptoms and half ended exercise sessions because of exhaustion, some of which could be related to central fatigue (271). Participants attained the targeted exercise intensity (50% of predicted HRR) in only 31% of sessions. Yet, only participants with mild to no disability were included in the study and body weight supported exercise was used when needed. This raises the possibility that only patients with less than mild disability would be able to reach intensity levels during walking that would provide a cardiovascular stimulus sufficient to achieve the neurologic benefit observed in animal models. In a recent study, our group revealed that only 28% of 61 people post-stroke with mild to moderate gait deficits were able to reach a walking intensity at or above the ATh when asked to walk at their maximal speed for 6 min (270). These patients were a mean of 13 ± 23 months post-stroke and time elapsed from the stroke did not influence ability to reach the ATh during the walking assessment (*p* = 0.5). Further, 58.3% were able to reach at most, a level that was 10% lower than the ATh and 73.3% at most, a level that was 15% lower than the ATh. Alternative modalities to walking, such as stationary cycling, may be better tolerated allowing higher intensity exercise but as demonstrated in two small randomized control studies may not result in improvements in ambulation (272, 273). Therefore, a training intensity that is likely feasible during a walking prescription and safe from a cerebral and cardiovascular point-of-view in the early-phases post-stroke is to start at light intensity and progress patients to a heart rate that is 10–15% lower than the heart rate that occurred at the ATh on a cardiopulmonary exercise stress test, or talk test; i.e., less than moderate intensity. Gradual intensity progression may also help to reduce risk of musculoskeletal issues owing to altered gait patterns.

#### Protecting the Brain From Excessive Elevation and Rapid Fluctuations in Mean Arterial Pressure

Beta-adrenergic blocking medication such as Metoprolol (Lopressor) and Atenolol (Tenormin) are prescribed to ~30–40% of people post-stroke (274). Beta-adrenergic blockade reduces cardiac output and CBF with exercise (162, 275). Therefore, patients who are prescribed a beta-blocker but are not at high risk of OH can be advised to perform aerobic exercise at a time when the medication is at maximum effect (i.e., ~2–4 h after oral administration depending on dose) as this may provide an extra level of cerebral protection. This would be expressly important for people with elevated resting BP. Another precaution for patients with borderline high resting BP in the early phases post-stroke is to prescribe exercise that engages a small amount of muscle mass (276), as greater muscle mass engagement is associated with a greater pressor response along with higher catecholamine concentrations that produce a higher BP response (277). For example, avoid prescribing exercise on modalities that engage both upper and lower extremities such as the elliptical machine and rowing ergometer (277). Another strategy to mitigate BP during exercise and for added cerebral protection is to avoid exercising in the morning. While not measured in stroke patients specifically, there can be early morning “surges” in BP and heart rate that have been observed in free-living conditions (278). In addition, early morning CA has been shown to be impaired in healthy people (62).

In healthy adults, there is a 5–10 s CA response time to a change in BP (279). Characterizing time delay has not been studied in humans following stroke. However, repeated exposure to rapidly changing blood flow that is not adequately controlled by CA has been associated with damage to the cerebral capillary bed (41). Therefore, precautions include avoiding exercise that results in rapid fluctuations in BP such as high intensity interval training and rowing in the early phases. The BP changes are likely too rapid to be immediately countered by CA which can result in pulsatile blood flow to the brain. Rowing results in both high concentrations of catecholamines likely related to the large muscle mass engaged and rapid fluctuations in cerebral perfusion pressure (277). In a study of 12 rowers, mean cerebral blood velocity increased to a peak of 88 ± 7 cm s^−1^ during the catch phase of the rowing cycle; this was also the phase that elicited the highest mean BP of 125 ± 14 mmHg. Also, avoiding sudden transitions in intensity by instituting a gradual ramping up or down in intensity during the warm up and cool down period to allow CA function time to respond is recommended.

In addition, avoid prescribing exercises that have an isometric component (like rowing) and that risk performing the Valsalva maneuver. There are multiple phases to the Valsalva maneuver, and each phase can have variable effects on cerebral perfusion pressure (280). CA in healthy individuals in some cases responds too slowly to counteract the sudden changes in BP related to the Valsalva maneuver. In the release phase for example, when there is a sudden release of the strain pressure, there is an elevated risk of cerebral hypoperfusion (281, 282). Early effects of stroke may further slow CA response time and have a deleterious effect. While this requires validation in stroke patients, it is recommended to avoid performing the Valsalva maneuver such as might occur in the catch phase of rowing.

## Conclusions

The timing of initiation and rate of progression of exercise and activity parameters are contingent on recovery of CA, resting BP, BBB function, hemorrhagic stroke parameters, ischemic penumbra, and cardiac-related complications. All physiological systems must be considered, including cardiac recovery that has to this point not been specifically considered. The early phases post-stroke are a dynamic and volatile time and careful application of mobilization and exercise therapy is required. Mobilization strategies need to mitigate the risk associated with orthostatic hypotension and prolonged standing, while exercise prescriptions need to be cognizant of the extent of BP elevation during exercise as well as the potential for post-exercise hypotension immediately thereafter. The strategies, precautions, and considerations, suggested herein, to safely mobilize patients are not resource intensive. Indeed, they are modifications to what is currently being practiced. We have also provided a more carefully considered screening criteria based on the literature.

Future studies may reveal a more complex association between timing of exercise interventions and recovery. Early interventions may prove beneficial to one parameter of stroke recovery such as cognition, but potentially harmful to another parameter. A profile of characteristics, including coexisting conditions such as diabetes, to identify patients that may benefit from a more rapid progression to higher intensity exercise should be undertaken. We have learned considerably from the AVERT study; however, before other studies of this magnitude and design are undertaken, it may be prudent to measure the acute effects of mobilization or aerobic exercise to help determine possible adverse effects. If detected, then strategies to counter the possible adverse effects of different intensities, duration, and modality during the early critical phases of the recovery continuum on CBF, BBB permeability, the ischemic penumbra, hematoma expansion, and other important physiological outcomes should be determined.

Effective use of early exercise and mobilization after stroke is currently limited by lack of data. Attempting to develop individualized approaches to exercise prescription are warranted but this requires a more holistic evaluation of the stroke survivor's overall fitness to exercise. Deeper and more comprehensive assessments will likely shed important new light in this important area of stroke recovery research.

## Author Contributions

SM drafted the manuscript. AR and BM critically revised and contributed to the conception and design of all parts of the manuscript. All others critically revised and contributed to the conception and/or design of parts of the manuscript.

### Conflict of Interest

The authors declare that the research was conducted in the absence of any commercial or financial relationships that could be construed as a potential conflict of interest.
