# Supplementary material for: Aerobic Training and Mobilization Early Post-stroke: Cautions and Considerations
Source: Front Neurol. 2019 Nov 15;10:1187. doi: 10.3389/fneur.2019.01187 (PMC6872678; doi:10.3389/fneur.2019.01187)
Supplement: Supplementary file 1 [file Presentation_1.pdf]

Aerobic Training and Mobilization Early Post-stroke: Cautions and Considerations

Supplementary Material Section

Estimated time of cerebral autoregulation recovery by stroke type

2.1.1 How long does cerebral autoregulation take to recover post-ischemic stroke?

Knowledge of the temporal profile of CA recovery would help in estimating when the brain is protected from the blood pressure fluctuations related to exercise or mobilization. A 2010 review paper identified 23 observational and randomized studies in which 16 studies measured CA 20-96 hours post stroke and 7 measured CA 7-458 days post-stroke. These studies used transcranial Doppler ultrasound with concomitant beat-to-beat blood pressure to characterize CA.[1] Overall, there was deterioration of CA from the 1st to the 5th day and recovery over 3 months. CA was impaired even after minor stroke and occurred bilaterally in many cases. Two studies demonstrated some degree of progressive impairment that the authors of the review suggested may affect penumbral salvage.[2, 3] These 2 studies demonstrated a worsening of the autoregulation index between the first 48 hours and 5 to 7 days post ischemic stroke. Worsening of CA impairment tended to occur mainly in relation to large infarctions. All 8 studies that measured dynamic CA showed evidence of impairment at baseline.[2-9] Since this review, Ma et al., measured dynamic CA in 67 consecutive post ischemic stroke patients and confirmed previous reports of impaired CA up to at least 10 days post event compared to controls.[10] Others have confirmed early impairment at 48 and 72 hours post stroke.[11, 12] Another subsequent study published in 2014 by Salinet et al., conducted repeated transcranial Doppler assessments at <72 hours, 2 weeks, and 1 and 3 months following infarction in 15 acute ischemic stroke and 22 healthy age- and sex-matched control subjects.[13] They reported significantly lower CBF velocity acutely post-stroke (<72 hours) relative to controls, and a reduction in CA index (worsening) that reached a nadir at 2 weeks post-stroke and returned to control levels within 3 months. Peterson et al. studied 28 patients with large-vessel ischemic stroke and demonstrated a transient CA impairment that was relegated to the affected side at a mean of 1.3±0.5 and 4.1±1 days, which normalized by 9.8±2.2 days after stroke.[14] The phase shift (smaller phase shift indicates poorer autoregulation) for the three time points were 29.6±10.5, 23.2±19.1 and 53.2±28.2 degrees respectively, with the first two but not the final measure being significantly lower than that measured in 29 healthy controls (47.9±16.8 degrees). Collectively, these studies suggest impaired CA at baseline with worsening in the first 1 to 2 weeks and a return to control levels at approximately 3 months post ischemic stroke (Figure 1). A limitation is that there are no measurements conducted between 1 and 3 months. However, one study demonstrated impaired CA in 71 small vessel disease patients at 48 hours post-stroke that was sustained at 6 months post event.[15] The authors speculate that impairment is a risk factor that leads to acute lacunar infarction rather than a result of acute infarction.

2.1.2 How long does cerebral autoregulation take to recover post-intracerebral hemorrhagic stroke?

Ma et al. performed serial measurements of dynamic CA in 43 patients with supratentorial ICH on days 1-2, 4-6, 10-12, and day 30 post stroke.[16] Dynamic CA was impaired bilaterally with worse autoregulation measured at days 10 to 12 and recovery occurring within a month. One study of 53 post-ICH patients showed bilaterally disturbed dynamic CA compared to healthy controls at days 4 to 6.[17] Ten ICH or traumatic brain injured patients demonstrated incomplete recovery at day 4 to 7[18] while others showed preservation at the acute stage and then deterioration in some patients on days 3 to 5 post-stroke.[19, 20] Two studies of 14 and 20 patient participants demonstrated no disturbance in CA in ICH measured within 24 hours of stroke[21, 22] but, as demonstrated in previous studies, patients may still deteriorate beyond this period starting at ~1.5 days.[23, 24] These studies suggest that there is little to no CA impairment at baseline, worsening at days 1.5 to 12, and then recovery at ~1 month post ICH (Figure). A limitation is that there is a dearth of measurements conducted between days 12 and 30.

2.1.3 How long does cerebral autoregulation take to recover post-subarachnoid hemorrhagic stroke?

The exact time course of CA recovery following SAH is not known. Rätsep et al. measured CA daily for 1 to 19 days in 52 patients; 60% demonstrated CA impairment that lasted between 1 to 10 days (mean 3.9 days).[25] Jaeger et al. demonstrated that people with more severe SAH exhibited initial impairment in dynamic CA which improved close to day 4.[26] In a group of patients with mixed severity SAH, dynamic CA was preserved in the first 2 to 3 days with subsequent deterioration in some patients.[27] In a longer term study, at 1 to 6 days following SAH, 4 of 12 patients had CA impairment (33.3%) and at days 7-13, 75% (9/12) had impaired CA.[28] More recently, Calviere et al. demonstrated CA impairment at baseline (within 4 days of SAH aneurysm rupture) and at day 7, and then returning to normal at day 14 in a cohort of 30 patients.[29] Therefore, the available evidence suggests a recovery profile that features some impairment through days 1 to 4 that can gradually deteriorate in some cases and then there is recovery by or after day 10 to 14 post-stroke (Figure).

2.3 Effect of comorbid conditions and age on cerebral autoregulation and blood brain barrier function

Cerebral autoregulation function in people with diabetes

Diabetes is a risk factor for stroke and represent roughly 30% of all people who have sustained a stroke.[30] [31] Hyperglycemia at the time of stroke increases mortality rate to 45% for those with diabetes and 78% in people without diabetes compared to ~30% mortality in people with stroke and normal glucose levels.[32] Diabetes results in extensive vascular damage and so it is not surprising that dynamic CA is impaired in people with type II diabetes, with or without cardiovascular autonomic neuropathy or microvascular complications.[33, 34] We therefore speculate that CA impairment may be more problematic among stroke patients with comorbid diabetes, and more research is needed on this subgroup. Cerebral protection during exercise may also be further compromised in people with stroke and diabetes as cerebral circulation is disturbed in response to exercise in people with diabetes alone (no stroke) when complicated by proliferative retinopathy.[35, 36] Other factors can affect CBF velocity such as very tightly

controlled blood pressure which results in lower CBF in people with diabetes.[37] Studies are needed to examine the effect of exercise on CBF in people with comorbid stroke and diabetes. In view of impaired CA in each condition alone, however, strategies to protect the brain during activity early post-stroke during CA dysfunction should be implemented and continued into the chronic phase of stroke.

Effect of age, diabetes, and hypertension on blood-brain barrier recovery

As well as the size of the infarct, the time course of recovery of BBB function in humans can be affected by age and comorbidities such as diabetes (hyperglycemia), and hypertension and should be considered when screening patients for initiating mobilization and aerobic exercise. Vascular aging renders the brain's vessels more susceptible to earlier and more severe BBB disruption.[38] People with diabetes and/or hyperglycemia can have profound BBB permeability levels following ischemic stroke increasing the risk of hemorrhagic transformation in patients treated with tPA.[39] For example, people with chronic hyperglycemia (defined by HbA1c >6.5%) had worse outcomes following ischemic stroke and tPA in one study[39] and several studies have suggested that a blood glucose level of ≥8.6mmol/L within the first 48 hours of stroke may be associated with poor outcomes.[40, 41] In pre-clinical studies of stroke, the presence of diabetes more than doubled the time for BBB recovery and the occurrence of hemorrhagic transformation.[42-44] Finally, hypertension triggers alterations that can lead to BBB dysfunction and increased permeability, suggesting that it is also an important pre-exercise screening criterion in the early phases post-stroke. Indeed, a link between BBB alterations, hypertension, and dementia can be drawn from animal models.[45-49] After ischemic stroke, hypertension increases infarct volume and white matter injury, and is associated with brain edema and cognitive deficits in animal models. This process is mediated in part by BBB breakdown.[45-47]

Estimated Time to Onset and Recovery of Cardiac Complications

3.3.1.1 Time of onset and recovery of systolic dysfunction

Banki et al. followed 173 patients with SAH over an 8 day period. Of those with LV dysfunction measured by ejection fraction (15%; n=25), and by regional wall-motion abnormality with normal LV function (13%; n=23), there was a trend toward a deterioration in cardiac function in the first two days after SAH, but by 8 days 66% of patients had recovered.[50] Of 277 consecutive SAH patients with no previously known ECG or echocardiographic abnormalities, 58 patients (21%) had wall motion abnormalities at admission and 16 (6%) developed new wall motion abnormalities on day 4.[51] Of the 58 patients with wall motion abnormalities on admission, only 14 (24%) had normal LV function on day 8. Also, 29 of 187 (16%) patients still alive and not discharged on day 8 had wall motion abnormalities. The authors point out that this study shows the dynamic nature of wall motion abnormalities.

3.3.2.1 Time of onset and recovery of arrhythmias

The risk of clinically significant cardiac arrhythmias is highest in the first 24 to 48 hours following stroke (Figure).[52, 53] For example, Fernández-Menéndez et al. reported that in a group of 332 patients admitted for ischemic stroke, intraparenchymal hemorrhagic stroke, and transient ischemic attack, 29.5% had significant cardiac arrhythmias during the hospital stay.[52] Specifically, ventricular tachyarrhythmias, supraventricular tachyarrhythmias, and complex ventricular ectopy were present in 27.1% of patients and bradyarrhythmia in 3.9% of patients.

The percentage of the occurrence of arrhythmias by time of monitoring on days 1, 2, and 3 post-stroke were 37%, 29% and 15% respectively. In a prospective study, Kalmunzer et al. reported that serious cardiac arrhythmias were detected in 25.1% of 501 patients in whom 92% were admitted with ischemic stroke.[53] The time course for onset of arrhythmia was 52% occurring within 12 hours and 74.4% within 24 hours after admission. Patients were monitored for a maximum of 72 hours. Atrial fibrillation can be present upon admission but can also develop a few hours to 3 days after stroke in the acute care setting.[54 55] One study reported that in a group of 346 consecutively admitted acute ischemic stroke patients, 34.4% were diagnosed with atrial fibrillation over 3 days of monitoring.[54] However studies vary widely, with the prevalence of atrial fibrillation reported to be as low as 2.5% over 3 days of monitoring in the acute phase post-stroke.[55]

3.3.3.2 Time of onset and recovery of myocardial injury

Kolin and Norris report that focal myocardial damage required at least 6 hours to develop after onset of the acute neurological event and was not observed after the second week.[56] Serial measures of troponin I in SAH reveal that troponin levels peaked between day 1 to 3 post-stroke and subsequently declined over 7 days.[57-60] For example, Naidech et al. measured troponin level in people with SAH and abnormal ECG or clinical signs or symptoms of potential cardiovascular dysfunction including elevated SBP (57.4%; 253 of 441 patients).[57] Of the 253 people tested, 172 (68%) had elevated troponin levels and were submitted to additional testing over 7 days. The peak median interval between SAH onset and peak troponin was 1.7 days (interquartile range 1.1 to 4.8 days) with no further peak levels detected by day 3. The mean troponin level declined over 7 days. Only 10 patients had a previous myocardial infarction. Yarlagadda et al. measured troponin on each of 2 study days in 300 patients post SAH. At a mean of 4.1±3.9 days post SAH, 22% of patients had an elevated level, and at a mean of 9.1±4.1 days post SAH, 19% had an elevated level. In ischemic stroke, 6.8% (50 of 738) of consecutive patients with no acute coronary syndrome had elevated serum cardiac troponin levels within 3 days of stroke.[61] Finally, in a group of 140 ischemic stroke patients, elevated troponin levels occurred in 6% (n=10) with the median time from stroke onset to elevation of 5.3 hours (range 1.5 to 20.4 hours), and the mean time to normalization was 12 hours (range 8 to 72 hours).[62]

Pre-clinical studies

4.2 Effects of aerobic exercise initiated at least six hours post-stroke

A series of preclinical studies by one group of authors examined the effects of a single 30-minute aerobic training session introduced very early (6 hours), early (24 hours), or relatively late (3 days) after reperfusion compared to non-exercise stroke controls.[63-66] Li et al. reported that 24 hours after exercise the very early exercise group (6 hours) had greater neuronal cell death than the non-exercise group.[63] This negative effect was not observed when exercise was initiated at 24 hours or 3 days. Very early exercise (6 hours) also resulted in greater infarct volumes and cell death than later exercise (24 hours or 3 days). Moreover, exercise initiated at 6 hours or 24 hours post reperfusion resulted in diminished brain oxidative metabolism (i.e. reduced energy production) and increased reactive oxygen species levels. Relatively late exercise (3 days) resulted in beneficial effects in all outcomes. Further, increased cell death after exercise initiated at 6 hours but not 3 days was associated with an exacerbation of hyperglycolysis.[65] The finding that only very early exercise exacerbated hyperglycolysis may in part explain the previously

cited findings of exacerbated brain injury during this very early phase, given that hyperglycolysis is associated with apoptosis. A separate study by the same group provides another possible apoptotic mechanism triggered in the very early post-stroke phase, with results indicating increased inflammatory cytokines following exercise initiated at 6 hours, but a reduction in cytokines when exercise was initiated at 3 days.[66]

Aerobic exercise initiated 24 to 48 hours post-stroke

A comprehensive systematic review published in 2014 identified 33 animal model studies investigating early phase aerobic exercise (*i.e.*, within 24–48 hours following the stroke).[67] The authors of this review report that no human studies satisfied the inclusion/exclusion criteria and thus only animal studies were included. The results from these studies suggest that either no change or a reduction in lesion volume occurs with an early exercise intervention compared to sedentary controls.[67] Eleven studies in the review reported on neuronal cell death, via markers of apoptosis, and found there were no adverse effects of exercise on when initiated as early as 24 hours post event. Further, 2 studies reported reduced oxidative damage and 3 studies revealed a reduction in inflammation when exercise was initiated in this early phase. Also, moderate intensity exercise was most effective for reducing lesion volume, increasing neurogenesis and decreasing inflammation, while higher intensity exercise for shorter duration increased pro-angiogenic proteins and genes in the area adjacent to the lesion and in the striatum.[68-71]

Subsequent to the 2014 review, Yang et al. measured motor, sensorimotor, and balance function in rats after 2 weeks of treadmill exercise training initiated at 24 hours post-stroke, and compared function to non-exercising stroke and exercising sham stroke control groups.[72] The exercising stroke group had greater improvement in coordinated locomotor function and spatial memory compared to the non-exercise stroke group, but there was no between-group difference in sensorimotor or vestibulomotor function.

Possible implications for aerobic exercise prescription in humans

Collectively, the preclinical data suggest that very early exercise (i.e. within 6 hours) may exacerbate brain injury, while early (*i.e.*, ~24 hours) and relatively late training (*i.e.*, >3 days) may be beneficial. Unfortunately, there is no formula that can map the rodent time frame post-stroke to a human time frame post-stroke. Knowledge translation between species continues to be a major challenge in early exercise and mobilization post-stroke. However, future pre-clinical studies are needed that examine the neuroprotective effect of exercise in the context of between-species biological clocks. Livingston-Thomas et al. suggest that exercise initiated in the first few days of the induced stroke may affect cell survival given that it takes approximately 1 to 3 days for the stroke to evolve and cells to die.[73-75] It is possible that compromised cells would have died at a later time point, independent of therapy, due to an early pruning effect whereby dysfunctional neurons are eliminated.[73-75] Further, there is no pre-clinical literature which examines the interaction between stroke characteristics (severity, type) or the presence of comorbidities such as diabetes, and the timing of exercise initiation or the initial exercise prescription. This would help to identify characteristics that predispose individuals to adverse effects from early exercise.

References

1. Aries MJH, Elting JW, De Keyser J, Kremer BPH and Vroomen PCAJ. Cerebral Autoregulation in Stroke: A Review of Transcranial Doppler Studies. *Stroke*. 2010;41:2697-2704.
2. Reinhard M, Roth M, Guschlbauer B, Harloff A, Timmer J, Czosnyka M and Hetzel A. Dynamic cerebral autoregulation in acute ischemic stroke assessed from spontaneous blood pressure fluctuations. *Stroke*. 2005;36:1684-1689.
3. Reinhard M, Wihler C, Roth M, Harloff A, Niesen W-D, Timmer J, Weiller C and Hetzel A. Cerebral autoregulation dynamics in acute ischemic stroke after rtPA thrombolysis. *Cerebrovasc Dis*. 2008;26:147-155.
4. Brodie F, Panerai R and Robinson T. dynamic Cerebral Autoregulation Is Impaired Acutely Following Ischaemic Stroke But Demonstrates Early Recovery: 15 Acute stroke: reorganization and recovery. *Cerebrovasc Dis*. 2009;27:210.
5. Eames P, Blake M, Dawson S, Panerai R and Potter J. Dynamic cerebral autoregulation and beat to beat blood pressure control are impaired in acute ischaemic stroke. *J Neurol Neurosur PS* 2002;72:467-472.
6. Dawson SL, Panerai RB and Potter JF. Serial Changes in Static and Dynamic Cerebral Autoregulation after Acute Ischaemic Stroke. *Cerebrovasc Dis*. 2003;16:69-75.
7. Dawson SL, Blake MJ, Panerai RB and Potter JF. Dynamic but not static cerebral autoregulation is impaired in acute ischaemic stroke. *Cerebrovasc Dis*. 2000;10:126-132.
8. Immink RV, Van Montfrans GA, Stam J, Karemaker JM, Diamant M and van Lieshout JJ. Dynamic cerebral autoregulation in acute lacunar and middle cerebral artery territory ischemic stroke. *Stroke*. 2005;36:2595-2600.
9. Atkins ER, Brodie FG, Rafelt SE, Panerai RB and Robinson TG. Dynamic cerebral autoregulation is compromised acutely following mild ischaemic stroke but not transient ischaemic attack. *Cerebrovasc Dis*. 2010;29:228-235.
10. Ma H, Guo Z-N, Jin H, Yan X, Liu J, Lv S, Zhang P, Sun X and Yang Y. Preliminary study of dynamic cerebral autoregulation in acute ischemic stroke: association with clinical factors. *Frontiers in neurology*. 2018;9.
11. Saeed NP, Panerai RB, Horsfield MA and Robinson TG. Does stroke subtype and measurement technique influence estimation of cerebral autoregulation in acute ischaemic stroke? *Cerebrovascular Diseases*. 2013;35:257-261.
12. Tutaj M, Miller M, Krakowska-Stasiak M, Piątek A, Hebda J, Łątka M, Strojny J, Szczudlik A and Słowik A. Dynamic cerebral autoregulation is compromised in ischaemic stroke of undetermined aetiology only in the non-affected hemisphere. *Neurologia i neurochirurgia polska*. 2014;48:91-97.
13. Salinet ASM, Panerai RB and Robinson TG. The Longitudinal Evolution of Cerebral Blood Flow Regulation after Acute Ischaemic Stroke. *Cerebrovascular Diseases Extra* 2014;4:186-197.
14. Petersen NH, Ortega-Gutierrez S, Reccius A, Masurkar A, Huang A and Marshall RS. Dynamic cerebral autoregulation is transiently impaired for one week after large-vessel acute ischemic stroke. *Cerebrovasc Dis*. 2015;39:144-150.
15. Guo Z-N, Xing Y, Wang S, Ma H, Liu J and Yang Y. Characteristics of dynamic cerebral autoregulation in cerebral small vessel disease: diffuse and sustained. *Scientific reports*. 2015;5:15269.
16. Ma H, Guo Z-N, Liu J, Xing Y, Zhao R and Yang Y. Temporal course of dynamic cerebral autoregulation in patients with intracerebral hemorrhage. *Stroke*. 2016;47:674-681.

17. Ma H, Guo Z-N, Sun X, Liu J, Lv S, Zhao L, Guo W, Jin H and Yang Y. Hematoma volume is a predictive factor of disturbed autoregulation after spontaneous intracerebral hemorrhage. *Journal of the neurological sciences*. 2017;382:96-100.
18. Schramm P, Klein KU, Pape M, Berres M, Werner C, Kochs E and Engelhard K. Serial measurement of static and dynamic cerebrovascular autoregulation after brain injury. *J Neurosurg Anesth*. 2011;23:41-44.
19. Reinhard M, Neunhoeffer F, Gerds TA, Niesen W-D, Buttler K-J, Timmer J, Schmidt B, Czosnyka M, Weiller C and Hetzel A. Secondary decline of cerebral autoregulation is associated with worse outcome after intracerebral hemorrhage. *Intensive Care Med*. 2010;36:264-271.
20. Oeinck M, Neunhoeffer F, Buttler K-J, Meckel S, Schmidt B, Czosnyka M, Weiller C and Reinhard M. Dynamic cerebral autoregulation in acute intracerebral hemorrhage. *Stroke*. 2013;44:2722-2728.
21. Gould B, McCourt R, Asdaghi N, Dowlatshahi D, Jeerakathil T, Kate M, Coutts SB, Hill MD, Demchuk AM and Shuaib A. Autoregulation of cerebral blood flow is preserved in primary intracerebral hemorrhage. *Stroke*. 2013;44:1726-1728.
22. Powers W, Zazulia A, Videen T, Adams R, Yundt K, Aiyagari V, Grubb R and Diringer M. Autoregulation of cerebral blood flow surrounding acute (6 to 22 hours) intracerebral hemorrhage. *Neurology*. 2001;57:18-24.
23. Lee Y-K, Tang S-C, Jeng J-S and Shieh J-S. Nonlinear analyses applied in cerebral autoregulation and blood flow changes in patients with acute intracerebral hemorrhage. *Biomedical Signal Processing and Control*. 2017;31:102-107.
24. Nakagawa K, Serrador JM, LaRose SL and Sorond FA. Dynamic cerebral autoregulation after intracerebral hemorrhage: A case-control study. *BMC neurology*. 2011;11:108.
25. Rätsep T and Asser T. Cerebral hemodynamic impairment after aneurysmal subarachnoid hemorrhage as evaluated using transcranial Doppler ultrasonography: relationship to delayed cerebral ischemia and clinical outcome. *J Neurosurg*. 2001;95:393-401.
26. Jaeger M, Schuhmann MU, Soehle M, Nagel C and Meixensberger J. Continuous monitoring of cerebrovascular autoregulation after subarachnoid hemorrhage by brain tissue oxygen pressure reactivity and its relation to delayed cerebral infarction. *Stroke*. 2007;38:981-986.
27. Budohoski KP, Czosnyka M, Smielewski P, Kasprowicz M, Helmy A, Bulters D, Pickard JD and Kirkpatrick PJ. Impairment of cerebral autoregulation predicts delayed cerebral ischemia after subarachnoid hemorrhage: a prospective observational study. *Stroke*. 2012;43:3230-3237.
28. Lang EW, Diehl RR and Mehdorn HM. Cerebral autoregulation testing after aneurysmal subarachnoid hemorrhage: the phase relationship between arterial blood pressure and cerebral blood flow velocity. *Crit Care Med*. 2001;29:158-163.
29. Calviere L, Nasr N, Arnaud C, Czosnyka M, Viguier A, Tissot B, Sol J-C and Larrue V. Prediction of delayed cerebral ischemia after subarachnoid hemorrhage using cerebral blood flow velocities and cerebral autoregulation assessment. *Neurocrit Care*. 2015;23:253-258.
30. Ivey FM, Ryan AS, Hafer-Macko CE, Garrity BM, Sorkin JD, Goldberg AP and Macko RF. High prevalence of abnormal glucose metabolism and poor sensitivity of fasting plasma glucose in the chronic phase of stroke. *Cerebrovasc Dis*. 2006;22:368-371.
31. Scott JF, Robinson GM, French JM, O'Connell JE, Alberti KG and Gray CS. Prevalence of admission hyperglycaemia across clinical subtypes of acute stroke. *Lancet*. 1999;353:376-377.
32. Candelise L, Landi G, Orazio EN and Boccardi E. Prognostic significance of hyperglycemia in acute stroke. *Arch Neurol*. 1985;42:661-663.
33. Mankovsky B, Piolot R, Mankovsky O and Ziegler D. Impairment of cerebral autoregulation in diabetic patients with cardiovascular autonomic neuropathy and orthostatic hypotension. *Diabetic Med* 2003;20:119-126.

34. Kim Y-S, Immink RV, Stok WJ, Karemaker JM, Secher NH and Van Lieshout JJ. Dynamic cerebral autoregulatory capacity is affected early in Type 2 diabetes. *Clin Sci (Colch)*. 2008;115:255-262.
35. Albert SG, Gomez CR, Russell S, Chaitman BR, Bernbaum M and Kong BA. Cerebral and ophthalmic artery hemodynamic responses in diabetes mellitus. *Diabetes Care*. 1993;16:476-482.
36. Lippera S, Gregorio F, Ceravolo M, Lagalla G and Provinciali L. Diabetic retinopathy and cerebral hemodynamic impairment in type II diabetes. *Eur J Ophthalmol*. 1997;7:156-162.
37. Kim Y-S, Davis SC, Truijen J, Stok WJ, Secher NH and Van Lieshout JJ. Intensive blood pressure control affects cerebral blood flow in type 2 diabetes mellitus patients. *Hypertension*. 2011;57:738-745.
38. Farrall AJ and Wardlaw JM. Blood–brain barrier: ageing and microvascular disease–systematic review and meta-analysis. *Neurobiol Aging*. 2009;30:337-352.
39. Masrur S, Cox M, Bhatt DL, Smith EE, Ellrodt G, Fonarow GC and Schwamm L. Association of Acute and Chronic Hyperglycemia With Acute Ischemic Stroke Outcomes Post-Thrombolysis: Findings From Get With The Guidelines-Stroke. *J Am Heart Assoc*. 2015;4:e002193.
40. Ribo M, Molina C, Montaner J, Rubiera M, Delgado-Mederos R, Arenillas JF, Quintana M and Alvarez-Sabín J. Acute hyperglycemia state is associated with lower tPA-induced recanalization rates in stroke patients. *Stroke*. 2005;36:1705-1709.
41. Fuentes B, Castillo J, San José B, Leira R, Serena J, Vivancos J, Dávalos A, Nuñez AG, Egido J and Díez-Tejedor E. The prognostic value of capillary glucose levels in acute stroke: the GLycemia in Acute Stroke (GLIAS) study. *Stroke*. 2009;40:562-568.
42. Reeson P, Tennant KA, Gerrow K, Wang J, Novak SW, Thompson K, Lockhart K-L, Holmes A, Nahirney PC and Brown CE. Delayed inhibition of VEGF signaling after stroke attenuates blood–brain barrier breakdown and improves functional recovery in a comorbidity-dependent manner. *J Neurosci* 2015;35:5128-5143.
43. Belayev L, Busto R, Zhao W and Ginsberg MD. Quantitative evaluation of blood-brain barrier permeability following middle cerebral artery occlusion in rats. *Brain Res*. 1996;739:88-96.
44. Chen J, Ye X, Yan T, Zhang C, Yang X-P, Cui X, Cui Y, Zacharek A, Roberts C and Liu X. Adverse effects of bone marrow stromal cell treatment of stroke in diabetic rats. *Stroke*. 2011;42:3551-3558.
45. Choi J, Cui Y and Kim B. Interaction between hypertension and cerebral hypoperfusion in the development of cognitive dysfunction and white matter pathology in rats. *Neuroscience*. 2015;303:115-125.
46. Fan Y, Lan L, Zheng L, Ji X, Lin J, Zeng J, Huang R and Sun J. Spontaneous white matter lesion in brain of stroke-prone renovascular hypertensive rats: a study from MRI, pathology and behavior. *Metab Brain Dis*. 2015;30:1479-1486.
47. Hom S, Fleegal MA, Egleton RD, Campos CR, Hawkins BT and Davis TP. Comparative changes in the blood-brain barrier and cerebral infarction of SHR and WKY rats. *Am J Physiol-Reg I*. 2007;292:R1881-R1892.
48. Popescu BO, Toescu EC, Popescu LM, Bajenaru O, Muresanu DF, Schultzberg M and Bogdanovic N. Blood-brain barrier alterations in ageing and dementia. *J Neurol Sci*. 2009;283:99-106.
49. Pelisch N, Hosomi N, Mori H, Masaki T and Nishiyama A. RAS inhibition attenuates cognitive impairment by reducing blood-brain barrier permeability in hypertensive subjects. *Current Hypertension Reviews*. 2013;9:93-98.
50. Banki N, Kopelnik A, Tung P, Lawton MT, Gress D, Drew B, Dae M, Foster E, Parmley W and Zaroff J. Prospective analysis of prevalence, distribution, and rate of recovery of left ventricular systolic dysfunction in patients with subarachnoid hemorrhage. *J Neurosurg*. 2006;105:15-20.
51. Van Der Bilt IA, Hasan D, Van Den Brink RB, Cramer MJ, Van Der Jagt M, Van Kooten F, Regtien JG, Van Den Berg MP, Groen RJ and Cate FJt. Time course and risk factors for myocardial dysfunction after aneurysmal subarachnoid hemorrhage. *Neurosurgery*. 2015;76:700-706.

52. Fernández-Menéndez S, García-Santiago R, Vega-Primo A, González Nafría N, Lara-Lezama LB, Redondo-Robles L, Montes-Montes M, Riveira-Rodríguez MC and Tejada-García J. Cardiac arrhythmias in stroke unit patients. Evaluation of the cardiac monitoring data. *Neurología (English Edition)*. 2016;31:289-295.
53. Kallmünzer B, Breuer L, Kahl N, Bobinger T, Raaz-Schrauder D, Huttner HB, Schwab S and Köhrmann M. Serious Cardiac Arrhythmias After Stroke: Incidence, Time Course, and Predictors—A Systematic, Prospective Analysis. *Stroke*. 2012;43:2892-2897.
54. Kallmünzer B, Breuer L, Hering C, Raaz-Schrauder D, Kollmar R, Huttner HB, Schwab S and Köhrmann M. A Structured Reading Algorithm Improves Telemetric Detection of Atrial Fibrillation After Acute Ischemic Stroke. *Stroke*. 2012;43:994-999.
55. Vingerhoets F, Bogousslavsky J, Regli F and Van Melle G. Atrial fibrillation after acute stroke. *Stroke*. 1993;24:26-30.
56. Kolin A and Norris JW. Myocardial damage from acute cerebral lesions. *Stroke*. 1984;15:990-993.
57. Naidech AM, Kreiter KT, Janjua N, Ostapkovich ND, Parra A, Commichau C, Fitzsimmons B-FM, Connolly ES and Mayer SA. Cardiac Troponin Elevation, Cardiovascular Morbidity, and Outcome After Subarachnoid Hemorrhage. *Circulation*. 2005;112:2851-2856.
58. Parekh N, Venkatesh B, Cross D, Leditschke A, Atherton J, Miles W, Winning A, Clague A and Rickard C. Cardiac troponin I predicts myocardial dysfunction in aneurysmal subarachnoid hemorrhage. *J Am Coll Cardiol*. 2000;36:1328-1335.
59. Deibert E, Barzilai B, Braverman AC, Edwards DF, Aiyagari V, Dacey R and Diringer M. Clinical significance of elevated troponin I levels in patients with nontraumatic subarachnoid hemorrhage. *J Neurosurg*. 2003;98:741-746.
60. Yarlagadda S, Rajendran P, Miss JC, Banki NM, Kopelnik A, Wu AH, Ko N, Gelb AW, Lawton MT and Smith WS. Cardiovascular predictors of in-patient mortality after subarachnoid hemorrhage. *Neurocrit Care*. 2006;5:102-107.
61. Ay H, Koroshetz WJ, Benner T, Vangel MG, Melinosky C, Arsava EM, Ayata C, Zhu M, Schwamm LH and Sorensen AG. Neuroanatomic correlates of stroke-related myocardial injury. *Neurology*. 2006;66:1325-1329.
62. Chalela JA, Ezzeddine MA, Davis L and Warach S. Myocardial injury in acute stroke. *Neurocrit Care*. 2004;1:343-346.
63. Li F, Shi W, Zhao EY, Geng X, Li X, Peng C, Shen J, Wang S and Ding Y. Enhanced apoptosis from early physical exercise rehabilitation following ischemic stroke. *J Neurosci Res*. 2016.
64. Li F, Geng X, Khan H, Pendy JT, Jr., Peng C, Li X, Rafols JA and Ding Y. Exacerbation of Brain Injury by Post-Stroke Exercise Is Contingent Upon Exercise Initiation Timing. *Front Cell Neurosci*. 2017.
65. Shen J, Huber M, Zhao EY, Peng C, Li F, Li X, Geng X and Ding Y. Early rehabilitation aggravates brain damage after stroke via enhanced activation of nicotinamide adenine dinucleotide phosphate oxidase (NOX). *Brain Res*. 2016;1648:266-276.
66. Li F, Pendy Jr JT, Ding JN, Peng C, Li X, Shen J, Wang S and Geng X. Exercise rehabilitation immediately following ischemic stroke exacerbates inflammatory injury. *Neurol Res*. 2017;39:530-537.
67. Austin MW, Ploughman M, Glynn L and Corbett D. Aerobic exercise effects on neuroprotection and brain repair following stroke: A systematic review and perspective. *Neurosci Res*. 2014;87:8-15.
68. Hu X, Zheng H, Yan T, Pan S, Fang J, Jiang R and Ma S. Physical exercise induces expression of CD31 and facilitates neural function recovery in rats with focal cerebral infarction. *Neurol Res*. 2010;32:397-402.
69. Yang Y-R, Chang H-C, Wang PS and Wang R-Y. Motor Performance Improved by Exercises in Cerebral Ischemic Rats. *J Motor Behav*. 2012;44:97-103.
70. Ma Y, Qiang L and He M. Exercise Therapy Augments the Ischemia-Induced Proangiogenic State and Results in Sustained Improvement after Stroke. *Int J Mol Sci*. 2013;14:8570.

71. F. M, H. S and Y. Y. The effects of early exercise on brain damage and recovery after focal cerebral infarction in rats. *Acta Physiol Scand*. 2011;201:275-287.
72. Yang L, Zhang J, Deng Y and Zhang P. The Effects of Early Exercise on Motor, Sense, and Memory Recovery in Rats With Stroke. *Am J Phys Med Rehabil*. 2017;96:e36-e43.
73. Garcia JH, Yoshida Y, Chen H, Li Y, Zhang ZG and Lian J. Progression from ischemic injury to infarct following middle cerebral artery occlusion in the rat. *Am J Pathol*. 1993;142:623-635.
74. Livingston-Thomas J, Nelson P, Karthikeyan S, Antonescu S, Strider M, Jeffers MS, Marzolini S and Corbett D. Exercise and Environmental Enrichment as Enablers of Task-Specific Neuroplasticity and Stroke Recovery. *Neurotherapeutics*. 2016;13:395-402.
75. Krakauer JW, Carmichael ST, Corbett D and Wittenberg GF. Getting Neurorehabilitation Right:What Can Be Learned From Animal Models? *Neurorehab Neural Repair*. 2012;26:923-931.
